# The Type VI Secretion Systems in Plant-Beneficial Bacteria Modulate Prokaryotic and Eukaryotic Interactions in the Rhizosphere

**DOI:** 10.3389/fmicb.2022.843092

**Published:** 2022-04-07

**Authors:** Emily N. Boak, Sara Kirolos, Huiqiao Pan, Leland S. Pierson, Elizabeth A. Pierson

**Affiliations:** ^1^Department of Horticultural Sciences, Texas A&M University, College Station, TX, United States; ^2^Department of Biology, Texas A&M University, College Station, TX, United States; ^3^Department of Embryology, Carnegie Institution for Science, Baltimore, MD, United States; ^4^Department of Plant Pathology and Microbiology, Texas A&M University, College Station, TX, United States

**Keywords:** T6SS, PGPB (plant growth-promoting bacteria), bacterivores, competition, *Pseudomonas*, rhizosphere ecology, GacS/GacA

## Abstract

Rhizosphere colonizing plant growth promoting bacteria (PGPB) increase their competitiveness by producing diffusible toxic secondary metabolites, which inhibit competitors and deter predators. Many PGPB also have one or more Type VI Secretion System (T6SS), for the delivery of weapons directly into prokaryotic and eukaryotic cells. Studied predominantly in human and plant pathogens as a virulence mechanism for the delivery of effector proteins, the function of T6SS for PGPB in the rhizosphere niche is poorly understood. We utilized a collection of *Pseudomonas chlororaphis* 30–84 mutants deficient in one or both of its two T6SS and/or secondary metabolite production to examine the relative importance of each T6SS in rhizosphere competence, bacterial competition, and protection from bacterivores. A mutant deficient in both T6SS was less persistent than wild type in the rhizosphere. Both T6SS contributed to competitiveness against other PGPB or plant pathogenic strains not affected by secondary metabolite production, but only T6SS-2 was effective against strains lacking their own T6SS. Having at least one T6SS was also essential for protection from predation by several eukaryotic bacterivores. In contrast to diffusible weapons that may not be produced at low cell density, T6SS afford rhizobacteria an additional, more immediate line of defense against competitors and predators.

## Introduction

The rhizosphere, the area surrounding plant roots that is directly influenced by root exudates and the associated microorganisms that comprise the rhizosphere microbiome, is an important source of plant-beneficial microorganisms (Bakker et al., 2012; Berendsen et al., 2012; Pascale et al., 2020). The rhizosphere niche is shaped by a complex community of bacteria, archaea, fungi, protists and viruses and the cooperative, competitive, and predatory interactions among members and with the plant root. Bacteria employ many different strategies to buffer and protect themselves from abiotic and biotic stresses, especially competition with other microorganisms and grazing by predators (Matz and Kjelleberg, 2005; Matz et al., 2005; Hibbing et al., 2010). These include multicellular behavior and formation of a biofilm community (Costerton, 1995; Yin et al., 2019) and the utilization of diverse secretion systems to deploy an arsenal of diffusible products enabling bacteria to defend or modify their “space” (Hibbing et al., 2010; Ghoul and Mitri, 2016; Stubbendieck and Straight, 2016; Dorosky et al., 2017; Granato et al., 2019). Many bacteria also have short-range mechanisms for the delivery of weapons or other products when in direct contact with sister cells or cells of other rhizosphere inhabitants of which the Type VI Secretion System (T6SS) is one of the best studied examples (Basler, 2015; Cianfanelli et al., 2016; Smith et al., 2020). It is becoming increasingly clear that T6SS mediate interactions important for competitive fitness in a variety of environments (Gallegos-Monterrosa and Coulthurst, 2021), although their roles in rhizosphere community dynamics and benefits to plant-beneficial bacteria is not well understood.

T6SS are needle-like injection systems and the encoding genes have been found in approximately 25% of Proteobacteria (Basler et al., 2013). T6SS have been shown to be important for the delivery of effector proteins into neighboring prokaryotic and eukaryotic cells and other intercellular interactions (Barret et al., 2011; Basler et al., 2013; Abby et al., 2016; Bernal et al., 2017b). Consequently, the primary focus of T6SS research has been on pathogenic bacteria and the role of T6SS in bacterial virulence and pathogenesis. A role for T6SS in virulence was first demonstrated using *Vibrio cholerae* and the bacterial predator *Dictyostelium discoideum* (Pukatzki et al., 2006). T6SS have been further implicated in virulence and killing of other eukaryotes such as *Caenorhabditis elegans*, an important bacterial predator and animal model organism (Vaitkevicius et al., 2006). T6SS have been shown to be important among human pathogens such as *Burkholderia pseudomallei, Pseudomonas aeruginosa, Vibrio cholerae*, and *Salmonella enterica* serovar Typhimurium and their T6SS have been linked directly to their virulence (Pukatzki et al., 2006; Basler et al., 2013; Aubert et al., 2016; Sana et al., 2016). T6SS are also important virulence factors among plant pathogens such as *Ralstonia solanacearum* and *Erwinia amylovora* (Tian et al., 2017; Asolkar and Ramesh, 2020). Although, T6SS have been well studied in pathogenic systems, much less information exists for their importance in the lifestyle of non-pathogenic organisms. Interestingly, many plant growth promoting bacteria (PGPB) also possess one or more T6SS (Loper et al., 2012; Marchi et al., 2013; Bernal et al., 2017a). Thus, it is likely that T6SS in PGPB may be involved in other functions related to their host-associated niche or plant beneficial activities.

Generally, T6SS are composed of 13–15 structural proteins divided into three interlocking structures: the intermembrane anchor, the baseplate, and the needle/sheath. The length of the entire structure has been shown to be determined by the width of the cell, which can measure up to 1 μm in length, allowing it to interact with both prokaryotes and eukaryotes (Basler et al., 2013; Abby et al., 2016; Bernal et al., 2017b; Santin et al., 2019). This needle is topped with a valine-glycine repeat protein G (VgrG) trimer, which is in turn topped by one proline-alanine-alanine-arginine (PAAR) repeat protein using hydrogen bonds (Shneider et al., 2013) and the PAAR protein acts as a sharpener enabling the end to penetrate neighboring cells (Ho et al., 2014; Basler, 2015). ClpV “recycles” the system by detaching the proteins and allowing them to reform elsewhere (Kapitein et al., 2013; Zoued et al., 2014). Other genes such as the serine/threonine kinase and phosphatase (*ppkA* and *pppA*) are associated with the genes encoding structural proteins, and PpkA and PppA are involved in the regulation of the firing of the system (Kulasekara and Miller, 2007; Chen et al., 2015).

A great deal of genetic diversity has been found among the operons encoding T6SS and many bacterial species have multiple T6SS-encoding operons that differ in terms of their genetic organization, the presence or absence of certain effectors, or their regulation (Chen et al., 2011, 2015; Loper et al., 2012; Spiewak et al., 2019). Efforts to characterize this diversity has led to T6SS-encoding operons being partitioned into five clades (Boyer et al., 2009; Bernal et al., 2017b). Characterization of T6SS using this clade system illustrates both the diversity in bacterial taxa having T6SS as well as the diversity of systems found within a single species. For example, *Burkholderia thailandensis* possesses five different T6SS and these systems have been shown to perform different roles (Schwarz et al., 2010). In *Pseudomonas*, T6SS may differ in terms of the type of stimuli that causes firing, e.g., contact-dependent firing or random firing. Contact-dependent firing is regulated by the signaling cascade TagQRST. This signaling cascade alerts the cell when damage has been caused to its membrane and triggers the formation of the T6SS in what is known as dueling behavior (Basler et al., 2013). In contrast, to fire randomly the TagQRST signaling cascade is not needed. Some *Pseudomonas* may have both dueling and random firing behavior, such as *P. fluorescens* Q287, which contains three T6SS clusters, one of which contains the TagQRST signaling cascade (Loper et al., 2012; Basler et al., 2013, this study). A direct method to predict T6SS function has not been established based purely on sequence analysis, but the genetic organization, effector types, and firing regulation are all possible determinants of functionality (Bernal et al., 2017b). Consequently, when more than one system is present within a species, it is possible that the systems perform different, non-redundant functions, increasing the repertoire of functionalities provided (Schwarz et al., 2010).

To gain a better understanding of the importance of T6SS for the rhizosphere lifestyle, we focused on *Pseudomonas chlororaphis* subsp. *aureofaciens* 30–84, a well-characterized plant PGPB that is an effective rhizosphere colonizer. Previous research demonstrated that production of phenazines, diffusible anti-microbial compounds, contributes to biofilm formation, rhizosphere competence, inhibition of fungal plant pathogens, plant disease suppression, and mediation of wheat seedling water and salt stress (Mazzola et al., 1992; Pierson and Thomashow, 1992; Maddula et al., 2006; Yu et al., 2018; Mahmoudi et al., 2019; Yuan et al., 2020). *P. chlororaphis* 30–84 also produces other diffusible weapons including hydrogen cyanide, several types of extracellular enzymes, and multiple R-tailocin particles, antibacterial proteins that resemble bacteriophage tails and target and kill other pseudomonads (Loper et al., 2012; Wang et al., 2013; Dorosky et al., 2017, 2018). The genome of *P. chlororaphis* 30-84 also encodes two genetically distinct T6SS (Loper et al., 2012, this study). With a diverse spectrum of diffusible protective mechanisms already at its disposal, we questioned whether the two T6SS, close-range defensive weapons were important for competitive rhizosphere fitness. We hypothesized, if both T6SS were functional, they may serve non-redundant functions that contribute to the ability of this PGPB to survive the rhizosphere niche and promote plant health. To test this hypothesis, we created mutants defective in one or both T6SS and used these derivatives to characterize the role of each T6SS in rhizosphere competence, bacterial competition, and protection from bacterivores. To examine the role of the T6SS in the context of the entire arsenal of competitive mechanisms, we used an existing collection of mutants including derivatives deficient in the production of phenazines (but not T6SS activity) and a global regulatory mutant *P. chlororaphis* 30–84 GacA (Chancey et al., 1999). The expression of T6SS-encoding genes in *P. chlororaphis* 30–84 are notably down regulated in *gacS* and *gacA* mutants (Wang et al., 2013), as observed in other *Pseudomonas* species (Hassan et al., 2010; Records and Gross, 2010; Chen et al., 2015). The GacS/GacA two component system also controls the production of secondary metabolites (including phenazines), bacteriocins, and extracellular enzymes in *P. chlororaphis* 30–84 (Wang et al., 2013) as observed in other PGPB (Heeb and Haas, 2001; Jousset et al., 2009). Having derivatives deficient in specific competitive mechanisms enabled us to distinguish the role of each T6SS from other mechanisms that contribute to competitive fitness and protection from bacterivores.

## Materials and Methods

### Bacterial Strains and Media

The bacterial strains and plasmids used in this study are described in Table 1. A spontaneous rifampin-resistant derivative of *P. chlororaphis* 30–84 was used in all studies, hereafter referred to as wild type (30–84 WT). *P. chlororaphis* and wheat rhizosphere test strains were grown at 28°C in the following media: Luria-Bertani (LB) (Fisher BioReagents, Hampton, NH, United States), AB minimal (2% glucose) (Chilton et al., 1974) amended with 2% casamino acids (AB + CAA) (CAA is from BD Bacto, San Jose, CA, United States) or King’s medium B (KMB) (King et al., 1954). *Escherichia coli* was grown at 37°C in LB medium, unless otherwise noted. *E. coli* and *Pseudomonas* strains were grown in liquid culture with agitation (200 rotations/min) or on solid medium (amended with agar at 15 g/l). Antibiotics were used in the following concentrations for *E. coli:* kanamycin (Km), gentamicin (Gm), carbenicillin (Cb), and 5-bromo-4-chloro-3-indolyl-β-D-galactopyranoside (X-gal) at 50, 15, 100, and 40 μg/ml, respectively; and for *P. chlororaphis:* Km, Gm, Cb, rifampicin (Rif), and cycloheximide at 50, 50, 100, 100, and 100 μg/ml, respectively.

**TABLE 1:** Bacteria strains and plasmids used in this study.

Strain	Description	References
***Pseudomonas***		
*P. chlororaphis* 30–84 WT	“Wild type,” Rif*^r^*	Pierson and Thomashow, 1992
*P. chlororaphis* 30–84 ZN	Phz^–^, Rif*^r^*, *phzB:lacZ* genomic fusion	Pierson et al., 1994
*P. chlororaphis* 30–84 GacA	Phz^–^ Rif*^r^* spontaneous *gacA* mutant	Chancey et al., 1999
*P. chlororaphis* 30–84 I/I2	*phzI:npt* and *csaI:uidA-Gm* genomic fusion, Gm*^r^*	Zhang and Pierson, 2001
*P. chlororaphis* 30–84 ΔTssA1	T6SS-1 mutant: Pchl3084_RS17705 replaced with Km*^r^* cassette	This study
*P. chlororaphis* 30–84 ΔTssA2	T6SS-2 mutant: Pchl3084_RS00080 replaced with Km*^r^* cassette	This study
*P. chlororaphis* 30–84 ΔTssA1/2	T6SS-1/2 mutant with Pchl3084_RS17705 replaced with Km*^r^* and Pchl3084_RS00080 replaced with Gm*^r^* cassette	This study
***Pseudomonas* rhizosphere colonizing, biocontrol strains**		
*P. protegens* Pf-5	Rhizosphere associated PGPB (formerly *P. fluorescens* Pf-5) with T6SS-encoding genes	Howell, 1979
*P. synxantha* 2-79	Rhizosphere associated PGPB (formerly *P. fluorescens* 2-79) without T6SS-encoding genes	Weller and Cook, 1983
*P. fluorescens* Q2-87	Rhizosphere associated PGPB with T6SS-encoding genes	Pierson and Weller, 1994
**Environmental and Plant Pathogenic Strains**		
*Pseudomonas putida* F1	Environmental isolate, without T6SS-encoding genes	https://genome.jgi.doe.gov/portal/psepu/psepu.home.html
*Pseudomonas syringae* pv. *tomato* DC3000	Plant pathogen with functional T6SS	Petnicki-Ocwieja et al., 2002
*Agrobacterium tumefaciens* C58	Plant pathogen with functional T6SS	Ma et al., 2014
*Pectobacterium carotovorum* subsp. *carotovorum*	Plant pathogen with T6SS-encoding genes	Lei et al., 2019
***Escherichia coli***		
*E. coli* DH5α	*F^–^recA1 endA1 hsdR17 supE44 thi-1 gyrA96 relA1*Δ(argF-lacZYA) Iq69 Φ80lacZΔM15λ^–^	GIBCO-BRL
*E. coli* HB101	F^–^ *hsds20*(r_*B*_^–^m_*B*_^–^)*supE44recA1 ara14 proA2 lacY1 galK2 rpsL20 xyl-5 mtl-5*λ^–^	GIBCO-BRL
*E. coli delta*B	*D. discoideum* food source	*Dictyostelium* Stock Center
*E. coli* OP50	*C. elegans* food source	Brenner, 1974
**Plasmids**	**Description**	**References**
pEX18Ap	Ap*^r^*	Hoang et al., 1998
pUC4K	Km*^r^*, Ap*^r^*	Grindley and Joyce, 1981
pUCP20Gm	Gm*^r^*, pUCp20 derivative containing constitutive promoter P*_*lac*_* with *Sma*I-flanked Gm*^r^* cassette inserted into the unique *Sca*I site within *bla*	Chiang and Burrows, 2003
pEX18A + TSSA2	pEX18A containing *tssA*-2 upstream and downstream sequences separated by a *Kpn*I restriction site	This study
pEX18A + TSSA2/KMR	pEX18A containing *tssA*-2 upstream and downstream sequences separated by a Km resistance cassette	This study
pEX18A + TSSA1/KMR	pEX18A containing *tssA*-1 upstream and downstream sequences separated by a Km resistance cassette	This study
pEX18A + TSSA2/GMR	pEX18A containing *tssA*-2 upstream and downstream sequences separated by a Gm resistance cassette	This study

*Ap^r^, Km^r^, Gm^r^, Rif^r^ indicate ampicillin, kanamycin, gentamicin, and rifampin, respectively.*

### Phylogenetic Analysis of Chromosomal Regions Containing Two Putative T6SS

**FIGURE 1:**
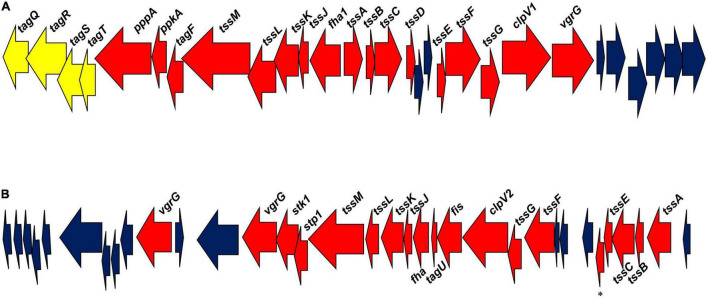
*Pseudomonas chlororaphis* 30–84 T6SS gene clusters. **(A)** The T6SS-1 cluster of genes. **(B)** The T6SS-2 cluster of genes. Red arrows designate genes encoding conserved proteins involved in T6SS structure and function. Blue arrows designate genes encoding hypothetical proteins. Yellow arrows designate genes encoding proteins associated with the TagQRST system in T6SS-1. *Refers to a gene encoding a T6SS protein with a PAAR domain.

*Pseudomonas chlororaphis* 30–84 genes annotated as encoding two T6SS are shown in Figure 1. We determined how these two T6SS compared to clades established by Boyer et al. (2009) and expanded by Bernal et al. (2017b). The amino acid sequences encoded by four highly conserved genes, *tssB, tssC, tssK*, and *tssM*, from each of the two *P. chlororaphis* 30–84 T6SS and the corresponding sequences from five species representing each clade were retrieved from the National Center for Biotechnology Information (NCBI) database and compared using BLASTp (Altschul et al., 1990). Based on levels of amino acid sequence identity, the *P. chlororaphis* 30–84 proteins were most similar to proteins in clades 3, 1.1, and 4A. The same *P. chlororaphis* 30–84 amino acid sequences were then compared to the corresponding amino acid sequences of 12 other plant-associated species belonging to clade 1.1, 3, or 4A. Also included were corresponding amino acid sequences from several biological control strains (Loper et al., 2012). The sequences were aligned using MUSCLE (MUltiple Sequence Comparison by Log-Expectation) through the program MEGA7 (Kumar et al., 2016). Once aligned, the Jones, Taylor, and Thorton (JTT) model in MEGA7 and bootstrap analysis with 1000 bootstrap replicates was used to build a maximum likelihood (ML) tree for each of the conserved genes. The program FigTree v1.4.4 (Rambaut, 2009; Gardner and Hall, 2013) was used to visually represent the ML trees.

### Generation of Single and Double T6SS Mutants

A derivative of *P. chlororaphis* 30-84 containing a *tssA-2* deletion mutation (ΔTssA2) was generated using the suicide vector pEX18Ap and using methods described previously (Hmelo et al., 2015). Briefly, DNA sequences (1,000 nucleotides [nt]) flanking the gene *tssA-2* were amplified by two-step PCR using the primer pairs TssA2KO-UP-F-*Eco*RI and TssA2KO-UP-R-*Kpn*I, and TssA2KO-DWN-F-*Kpn*I and TssA2KO-DWN-R-*Hin*dIII, respectively (Supplementary Table S1). Amplification using primers TssA2KO-UP-F-*Eco*RI and TssA2KO-DWN-R-*Hin*dIII and using the product of the previous PCRs as a template resulted in a construct that contained the upstream fragment separated from the downstream fragment by a *Kpn*I restriction site. This fragment was ligated into the *Eco*RI and *Hin*dIII restriction enzyme sites in the multiple-cloning region of pEX18a to create plasmid pEX18A + TSSA2. A kanamycin resistance cassette with its promoter was obtained via PCR amplification using pUC4K as the template and the primers TssA2KO-UP-R-*Kpn*I and TssA2KO-DWN-F-*Kpn*I and ligated between the upstream and downstream fragments at the *Kpn*I site in pEX18Ap. The final construct (pEX18A + TSSA2/KMR) was transformed into *E. coli* DH5α, and transformants were selected on LB amended with Km and Xgal. After conjugation, double-crossover mutants into *P. chlororaphis* were obtained by counterselection on LB amended with Rif, Km, and 6% sucrose and confirmed using PCR primers specific to the internal regions of *tssA-2.* PCR was performed using GoTaq^®^ Green Master Mix (Promega, Madison, WI, United States) according to manufacturer recommendations. *E. coli* transformation and *P. chlororaphis* conjugation were performed as described previously (Pierson and Thomashow, 1992; Wang et al., 2012).

A derivative of *P. chlororaphis* 30-84 containing a *tssA-1* deletion mutation (ΔTssA1) was generated using the suicide vector pEX18Ap. Briefly, DNA sequences upstream (∼1200 nt) and downstream (∼1,100 nt) flanking *tssA-1* were amplified via PCR using the primer pairs TssA1KO-repliQa-UP-F and TssA1KO-repliQa-UP-R, and TssA1KO-repliQa-DWN-F and TssA1KO-repliQa-DWN-R, respectively (Supplementary Table S1). The kanamycin resistance cassette with its promoter was amplified via PCR using pUC4K as the template and primers TssA1KO-repliQa-KmR-F and TssA1KO-repliQa-KmR-R. The final construct (pEX18A + TSSA1/KMR) was obtained using repliQa HiFi Assembly according to the manufacturer (“RepliQa HiFi ToughMix | Superior Speed and Inhibitor Tolerance | Quantabio, 2021”). DH5α transformants were selected on LB amended with Km and Xgal. *P. chlororaphis* double-crossover mutants were obtained by counterselection on LB amended with Rif, Km and 6% sucrose and confirmed using PCR primers specific to the internal regions of *tssA-1.*

To generate the double mutant ΔTssA1/2, a gentamicin resistant cassette with its promoter was amplified using the plasmid pUCP20Gm as the template and the primer pairs DoubleKO-GnR + A2UP-R and DoubleKO-GnR + A2DWN-F. The plasmid pEX18A + TSSA2 was digested with *Kpn*I. The final construct containing the gentamicin resistance cassette (pEX18A + TSSA2/GMR) inserted at the *Kpn*I site was obtained using repliQa HiFi Assembly. The plasmid pEX18A + TSSA2/GMR was transformed into *E. coli* DH5α and transformants were selected on LB amended with Gm and Xgal. *P. chlororaphis* double-crossover mutants in ΔTssA1 were obtained by counter selection on LB amended with Rif, Km, Gm and 6% sucrose and confirmed using PCR primers specific to the internal regions of *tssA-1* and *tssA-2.*

### Growth in Planktonic Culture and Surface Attached Biofilms

The strains 30-84 WT, ΔTssA1, ΔTssA2, and ΔTssA1/2 were grown in LB medium at 28°C with agitation and the optical density (OD_620_) was measured at one-hour intervals until 8 h and then at two-hour intervals between 24 and 30 h The experiment was performed with two biological replicates and repeated three times.

Surface attached biofilm formation was quantified via 96-well microtiter assay routinely used in our lab (Maddula et al., 2006) with slight modifications. Briefly, pre-cultures were grown in LB medium (28°C, with agitation, 16 h). These cultures were resuspended in fresh LB medium and grown to a final cell density of OD_620_ = 0.8. Each strain (1.2 μL) was inoculated into 120 μL AB + CAA in separate wells of a 96 well polystyrene cell culture plate (Corning Inc., Corning, NY, United States). The plate was incubated at 28°C for 72 h without agitation in a sealed container to minimize evaporation. Unattached cells were removed by inversion of the plate with vigorous tapping. The adherent bacteria were fixed to the plate (20 min, 50°C) and stained (1 min, 150 μL of 0.1% crystal violet). Excess stain was removed by inversion of the plate followed by two washings with sterile distilled water. The adherent cells were decolorized (to release the dye) with a 20% acetone/80% ethanol solution (200 μL, 5 min, room temperature). A sample (100 μL) of each well was transferred to a new 96-well plate and the amount of dye (proportional to the density of adherent cells) was quantified (OD_540_). The experiment was performed with two biological replicates (separate colonies) and five technical replicates and repeated three times.

### Rhizosphere Colonization and Persistence Assay

We used repeated planting/harvest cycles to evaluate rhizosphere persistence as described previously (Mazzola et al., 1992). The assay was performed using methods described previously (Dorosky et al., 2017) with minor modifications. Soil used for rhizosphere experiments was a Pullman clay loam collected from the USDA-ARS, Bushland, TX dryland wheat plots at a depth of 1–15 cm. Prior to use in experiments, it was necessary to sieve (2 mm mesh) and mix the soil with sand (soil:sand, 2:1, v:v) to facilitate drainage as described previously (Mahmoudi et al., 2019). The soil-sand mix is hereafter referred to as soil. The hard red winter wheat cultivar TAM 304 (Rudd et al., 2015) was used for all rhizosphere studies.

Bacteria were grown overnight with antibiotic selection, washed twice with sterile water, and resuspended in sterile water at a final concentration of 1 × 10^9^ CFU/mL. Inoculum was added to either steam-sterilized soil (autoclaved twice: 121°C, 15 psi, 45 min, 24 h pause between cycles) or untreated (field) soil. Final bacterial concentrations were adjusted to 10^6^ CFU/g by dilution using sterile water, adding the diluted suspension to soil (20 mL solution per 500 g), and mixing thoroughly daily for four days. Soil was then added to clean conical plastic growth tubes (Ray Leach Cone-tainers, 4 cm diameter, 21 cm height).

Wheat seeds (TAM 304) were surface disinfested by incubation in 10% bleach (10 min.) and then washed with sterile water (five times for 1 min. each). Disinfested seeds were pregerminated on germination paper for 48 h. The seedlings were planted in the growth tubes four days after the soil was inoculated with bacteria. A total of 50 plants were sown at the start of the experiment. Plants were grown on a light bench (8 h:16 h dark/light cycle, room temperature) and given sterile water (10 mL) every five days. After 20 days of growth, 10 of the 50 plants from each treatment were randomly selected and harvested and rhizosphere populations determined. The unharvested plants (remaining 40 of the 50 plants/treatment) were removed individually from their containers, the shoot system was excised and discarded, and the soil and root system were transferred to a clean paper cup, mixed by shaking, and returned to the conical growth tube from which they were obtained. This soil was then replanted with disinfested, pregerminated wheat seeds to initiate the second 20-day planting/harvest cycle. At each harvest, 10 of the remaining plants from each treatment were harvested and rhizosphere populations determined. The planting to harvest cycle was repeated for a total of five cycles. The entire experiment was repeated three times.

### *In vitro* and Rhizosphere Competition Assays

Competitive fitness assays compared the populations of competitors grown separately and in 50:50 mixed cultures *in vitro*. Briefly, bacterial strains were grown overnight in LB at 28°C with agitation (200 rpm), harvested, washed, and resuspended in fresh medium (cell densities were adjusted to OD_620_ = 0.5) before creating the single strain or mixed starting cultures. Mixed cultures were prepared using equal volumes of competitors. A total of 10 μl per treatment was placed onto ∼ 1 cm^2^ pieces of nitrocellulose filter paper on LB plates, and plates were incubated at 28°C for 5 h. Nitrocellulose papers then were transferred separately to sterile tubes containing 1 mL sterile water (sufficient to cover filter paper), and cells were collected by vortexing for 30 s twice with a 5 min. rest in between. Bacterial populations were enumerated via serial dilution plating on LB after 48 h. *P. chlororaphis* 30–84 and derivative appear bright orange on plates making them easy to distinguish from competitors. A competitive ratio was calculated by standardizing the population size of each strain in mixture to the population size of that strain without a competitor. The experiment was performed with five biological replicates and repeated three times.

Competitive fitness assays comparing the growth of competitors grown separately and in 50:50 mixed cultures in the wheat root rhizosphere were performed similar to previous methods (Dorosky et al., 2017). The inoculum was prepared as described for the *in vitro* assay, but the final total cell density used to inoculate seeds was 10^9^CFU/ml (OD_620_ = ∼1.0). Wheat seeds were surface disinfested and pregerminated (as described above) and then suspended in bacterial inoculum for 10 min. The seeds were sown into steam-sterilized soil (prepared as above). Plants were grown and maintained as above. After 28 days, the entire root system and loosely adhering soil was transferred to a sterile plastic tube (15 mL), immersed in 5 mL of sterile water and sonicated and vortexed three times (10 s each). Serial dilutions were plated onto LB amended with cycloheximide and bacterial populations quantified after 48 h. The roots were dried for 48 h at 65°C and populations were standardized to root dry weight. Competitive ratios were calculated as above. The experiment was performed with 8–10 replicates/treatment and repeated three times.

### Predator-Prey Studies

#### Dictyostelium discoideum

*Dictyostelium discoideum* strain AX2 was purchased from the *Dictyostelium* stock center (Fey et al., 2013). *D. discoideum* cells were grown in SIH medium (Formedium, Hunstanton, United Kingdom) with agitation as described previously (Brock and Gomer, 1999; Rijal et al., 2019). *D. discoideum* cells were collected by centrifugation (500 × *g*, 3 min.), resuspended/washed in fresh SIH twice, and resuspended at a final concentration of 1 × 10^6^ cells/mL in SIH (determined via direct counts using a hemocytometer). *D. discoideum* cells (1 mL/well) were added to 24-well plates (Cat. #353047, Corning, NY, United States), allowed to adhere for 30 min., and then the liquid medium was replaced with low nutrition PBM or high nutrition HL5 (Phillips and Gomer, 2012).

Bacterial cultures were grown in LB media for 16 h, and bacterial cells were collected by centrifugation (2,000 × g, 1 min.) and resuspended in PBM. Bacterial cultures were then standardized to a low cell density (OD_600_ = 0.1) and added to the wells containing *D. discoideum* cells. Bacteria used as prey in the feeding assay included 30–84 WT, ΔTssA1, ΔTssA2, ΔTssA1/2, and *P. chlororaphis* derivatives 30–84 GacA, 30–84 I/I2, or 30–84 ZN. *D. discoideum* cells growing without bacteria as a food source or with *E. coli*ΔB (used in lab as a preferred prey source) were used as controls. After 24 h, the mixed cultures were slowly resuspended with a pipettor to detach the *D. discoideum* cells from the wells and 200 μL from each well was transferred into a 96-well microtiter-plate suitable for microscopy (#160822/1, ibidi, Martinsried, Germany). The *D. discoideum* cells were left to adhere for 30 min. Differential Interference Contrast (DIC) images were obtained using a Nikon Ti2 Eclipse Microscope (40X, 100X oil). Another 200 μL from each well was used for serial dilutions to determine bacterial populations.

To measure Contact Site A protein (CsA) and Discoidin I levels in D. discoideum cells, the phagocytosis assay was performed as above. After 24 h, the supernatant was removed from each well and 200 mL of 2X SDS sample buffer was added to each well. The cells were collected by pipetting up and down repeatedly. The collected material was then heated to 95°C for 5 min. Samples were electrophoresed and blotted as described previously (Bakthavatsalam et al., 2008) except that the blots were blocked in 5% non-fat skim milk (Difco, Franklin Lakes, NJ, United States) in PBST [Phosphate Buffered Saline (pH 7.4) + 0.1% Tween-20] for 1 h and stained as previously described (Rijal et al., 2019) with either 1:500 #20-121-1-s anti-CsA (Developmental Studies Hybridoma Bank, Iowa City, IA, United States), 1:500 #80-52-13-s anti-Discoidin I (Developmental Studies Hybridoma Bank, Iowa City, IA, United States) or 1:1000 #13E5 beta-Actin (Cell Signaling Technology, Danvers, MA, United States) and then using a a secondary antibody 1:2500 #715-036-150 peroxidase-conjugated donkey anti-Mouse IgG (Jackson ImmunoResearch, West Grove, PA, United States) or 1:2500 #711-036-152 peroxidase-conjugated donkey anti-Rabbit IgG (Jackson ImmunoResearch, West Grove, PA, United States). Staining was detected with Supersignal West Pico PLUS Chemiluminescent Substrate for 10 min. (Cat # 34087, Thermo, Waltham, MA, United States). Images of the membranes were taken using a BioRad ChemiDoc XRS system and quantified using the Image Lab software (Bio-Rad, Hercules, CA, United States). Band intensities were normalized to the corresponding total actin band intensity.

To determine the effect of predation on bacterial fitness, a bacterial clearing assay was performed as described previously (Phillips and Gomer, 2010). Briefly, bacterial cultures and *D. discoideum* cells were grown and collected as described above but the final concentration of *D. discoideum* was adjusted to 500,000 cells/mL. 100 μL of each bacterial culture were spread onto SM/5 (pH = 6.5) medium plates and 10 μL of *D. discoideum* was then transferred to the center of the plate. Plates were incubated at 22°C for 5 days and the diameter of the zone of clearing was measured after 2 and 5 days. The experiment was repeated using four biological replicates.

#### Tetrahymena thermophila

For the feeding assay, *T. thermophila* CU427 (*Tetrahymena* Stock Center, Cornell University, Ithaca, NY, United States) were grown according to a previous protocol with minor modifications (Wilson et al., 1999). Briefly, *T. thermophila* was grown in PPYS (2% proteose peptone, 90 μM sequestrene, 0.2% yeast extract) liquid medium overnight with agitation (200 rpm, 30°C) and then transferred to 200 mL fresh media. Populations were adjusted to 3 × 10^5^ cells/20 mL via direct population counts using a hemocytometer and Leitz (Epivert) microscope (100X). Bacterial cultures were grown in LB for 16 h, and bacterial cells were collected by centrifugation (3,000 × *g*, 15 min) and washed with an equal amount of sterile water. Bacterial cultures were then standardized to a low cell density (OD_620_ = 0.1). Bacteria used as prey in the feeding assay included 30–84 WT, ΔTssA1, ΔTssA2, ΔTssA1/2, 30–84 GacA, 30–84 I/I2, or 30–84 ZN. *T. thermophila* without prey bacteria was used as a control. Bacterial and *T. thermophila* cultures were mixed (5 mL, 20 mL, respectively) and grown in 50 mL tubes with agitation (200 rpm, 27°C). After 4 h and 24 h, *Tetrahymena* populations in mixed cultures were enumerated via direct counts using a hemocytometer.

For the mating assay, *T. thermophila* with different germlines, CU427 and CU330, were selected. These were selected as they are non-self-cells of different germlines and will reproduce together (Cervantes et al., 2013). CU427 and CU330 (*Tetrahymena* Stock Center, Cornell University) were grown in PPYS liquid media overnight (200 rpm, 30°C) and collected via centrifugation (3,000 × *g*, 10 min.) and washed in an equal amount of 10 mM Tris Buffer (pH = 7.4) twice. *T. thermophila* were grown overnight (200 rpm, 30°C) in 10 mM Tris Buffer to induce starvation and then populations were standardized to a cell density of 1.5 – 2 × 10^5^/10 mL (via direct counts). Bacterial cultures were prepared as described above and standardized to OD_620_ = 0.1. *T. thermophila* CU427 and CU330 cultures were mixed in equal amounts (10 mL each) with 1 mL of the bacterial culture. After 4 h the treatments were viewed under the Leitz (Epivert) microscope, and the frequency of mated cell pairs (number of mated pairs/total number of observations) determined. Mating is defined by the joining to two *T. thermophila* cells vs. cells that remain single. This experiment was repeated three times.

#### Caenorhabditis elegans

*Caenorhabditis elegans* N2 hermaphrodites (*Caenorhabditis* Genetics Center, University of Minnesota, Minneapolis, MN, United States) were partially synchronized by allowing the nematodes to crowd a Nematode Growth Media (NGM) plate (Brenner, 1974) and consume all of their food source. Once the eggs produced on this plate hatched to stage L1 they were removed to a fresh NGM plate with *E. coli* OP50 for food and allowed to grow (20°C) to stage L4. Once mature, five L4 adult nematodes were selected and placed onto new NGM plates inoculated with one of the different prey bacteria: *E coli* OP50 (control), 30–84 WT, ΔTssA1, ΔTssA2, ΔTssA1/2, 30–84 GacA, 30–84 I/I2, or 30–84 ZN. Nematodes were allowed to lay eggs for 1 h (at room temperature) and then transferred to a new prey-containing plate. This transfer protocol was repeated until there were four plates per treatment, with the original nematodes being removed from the fourth plate. The plates were observed every 24 h for 72 h. The numbers of immature and mature nematodes were enumerated, and the percentage of adult nematodes calculated. Images of the plates were taken after 72 h using a Zeiss Stemi SV11 scope (26X magnification) and a Hamamatsu ImagEM EM-CCD camera. Bacterial clearing was estimated from total space occupied by *C. elegans* at the end of 72 h. Experiments were replicated three times.

### Statistical Analyses

All data presented are the mean ± the standard error from at least two experiments. Unless otherwise indicated, multiple comparisons were analyzed (α = 0.05) using ANOVA and either Tukey HSD or Student’s *t* tests and significant differences (*P* < 0.05) are indicated by lowercase letters. All data were analyzed using JMP Version 16 Software (SAS Institute Inc., Cary, NC, United States).

## Results

### *Pseudomonas chlororaphis* 30–84 Has Two Putative T6SS

*Pseudomonas chlororaphis* 30–84 contains two separate T6SS gene clusters, T6SS-1 and T6SS-2 (Figure 1), both of which contain genes encoding at least 12 of the 13 conserved T6SS proteins (*tssA-tssM*). These proteins combine into subunits that make the three structures necessary for T6SS formation: the intermembrane structure (composed of the proteins TssJ, TssL, and TssM); the baseplate structure (composed of TssE, TssF, TssG, and TssK); and the sheath and needle-like structure (TssB, TssC and Hcp/TssD) with TssA coordinating the assembly of the final structure (Planamente et al., 2016; Zoued et al., 2017). The gene encoding Hcp is not present in the T6SS-2 cluster, but an additional putative *hcp* gene is found elsewhere in the genome. The needle-like structure is topped with a valine-glycine repeat protein G (VgrG/TssI) trimer that in most cases is associated with effector proteins (Ho et al., 2014). The proline-alanine-alanine-arginine (PAAR) repeat protein sits atop VgrG and acts as a sharpener enabling the end to penetrate neighboring cells (Shneider et al., 2013; Ho et al., 2014; Basler, 2015). Both T6SS-1 and T6SS-2 have genes encoding putative VgrG proteins associated with their clusters (one and two genes, respectively), and an additional seven putative VgrG-encoding genes are found elsewhere in the genome. Only the T6SS-2 contains a gene encoding PAAR protein in its cluster, with nine more putative PAAR protein-encoding genes occurring elsewhere in the genome. ClpV (TssH) is involved in recycling the system (Kapitein et al., 2013; Zoued et al., 2014) and is found in both T6SS clusters. Hcp, VgrG, and PAAR proteins are associated with the delivery of effectors (Ho et al., 2014) and effectors are frequently identified based on their proximity to the encoding genes in the genome (Spiewak et al., 2019). The locations of these genes in the genome were used to search for putative effectors associated with both T6SS.

The two T6SS differ in organization. In T6SS-1, the structural genes are divergently transcribed whereas in T6SS-2 the genes are transcribed in the same direction. Additionally, the genes encoding the regulation cascade system TagQRST, shown previously to be responsible for contact-dependent firing used in T6SS dueling (Basler et al., 2013) are found only within T6SS-1 gene cluster, suggesting this system may be fired in a contact-dependent manner. Genes encoding a serine/threonine kinase (PpkA) required to phosphorylate the protein Fha for the dueling signal to be received and induce T6SS formation and a serine/threonine phosphatase (PppA) required to dephosphorylate Fha allowing the T6SS to be dismantled and recycled by ClpV (Casabona et al., 2013; Ho et al., 2014) are also present in T6SS-1. Although the T6SS-2 cluster lacks the Tag regulatory cascade, it contains genes encoding a serine/threonine kinase (Stk1), Fha, and a serine/threonine phosphatase (Stp1), suggesting random firing as seen in other systems (Basler and Mekalanos, 2012). Based on the differences in the organization and regulation of these systems, we hypothesized that they respond to different stimuli and potentially serve non-redundant functions.

We compared the two *P. chlororaphis* 30-84 T6SS clusters to the genetic clades established by Boyer et al. (2009) and expanded by Bernal et al. (2017b). The predicted amino acid sequences of four highly conserved structural genes, *tssB, tssC, tssK*, and *tssM* from T6SS-1 and T6SS-2 and the corresponding sequences from five species representing each clade were compared using BLASTp. Based on the levels of amino acid sequence identity, the proteins in T6SS-1 were most similar to clade 3, whereas the amino acid sequences in T6SS-2 were similar to both clades 1.1 and 4A. The predicted amino acid sequences of these genes in T6SS-1 and T6SS-2 were then compared to corresponding sequences from 12 other plant-associated species belonging to clade 1.1, 3, or 4A. Maximum likelihood trees were constructed for each protein and confirmed that the amino acid sequences in T6SS-1 group aligned optimally with clade 3. Clade 3 includes T6SS from a wide variety of genera, including the *P. aeruginosa* T6SS involved in contact-dependent dueling (Basler et al., 2013). The amino acid sequences in T6SS-2 predominately align with clade 1.1 (although a single strain belonging to 4A clustered nearby), which includes T6SS from many different pseudomonads such as *P. fluorescens* strains that appear to be random firing systems (Bernal et al., 2017b). The maximum likelihood tree for TssB (also used for clade analysis by Bernal et al., 2017b) appears in Supplementary Figure S1.

### Growth of *Pseudomonas chlororaphis* T6SS Mutants

To study the function of each T6SS, mutants were generated to disrupt T6SS assembly via deletion of *tssA* in each system and double mutants generated in both systems. In planktonic culture, there was no difference in the growth rates of 30–84 WT or the single T6SS mutants (ΔTssA1 and ΔTssA2), although the double mutant (ΔTssA1/2) consistently grew somewhat slower and reached a slightly lower cell density after 30 h (Supplementary Figure S2A). However, in surface-attached biofilms, population levels of 30-84 WT and the single or double T6SS mutants were no different after 72 h (Supplementary Figure S2B).

**FIGURE 2:**
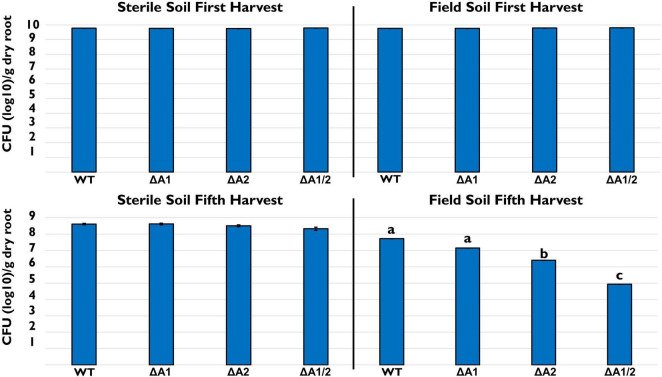
Rhizosphere persistence over repeat harvests. Bacterial populations (log10 CFU/g root dry weight) in sterile and field soil after the first and fifth harvest. Strains tested included 30–84 WT, the single T6SS mutants ΔTssA1 and ΔTssA2, and the double mutant ΔTssA1/2. Data are the mean and standard errors (bars may be too small to see for some treatments) from three replicate experiments (*n* = 10/per replicate). Lettering indicates significant differences. Data were analyzed using a one-way ANOVA and Tukey’s tests and significant differences are indicated, *p* < 0.05.

We also looked at the ability of 30–84 WT, ΔTssA1, ΔTssA2, and ΔTssA1/2 to colonize and persist in the wheat rhizosphere after multiple plant/harvest cycles in steam-sterilized and untreated field soil. No differences in the rhizosphere populations of strains were observed at the end of one harvest cycle in either sterile or field soil (Figure 2), indicating no loss in colonizing ability by the mutants. In the steam-sterilized soil, although the rhizosphere populations were slightly smaller at the end of five plant harvest cycles compared to the first harvest, there were still no statistical differences among treatments. In contrast, in natural soil the rhizosphere populations of ΔTssA1/2 were significantly reduced compared to 30–84 WT, whereas populations of ΔTssA2 were intermediate (Figure 2). These data suggested that having a T6SS is not necessary for rhizosphere colonization but is important for competitive persistence.

### Bacterial Competition Assays

**FIGURE 3:**
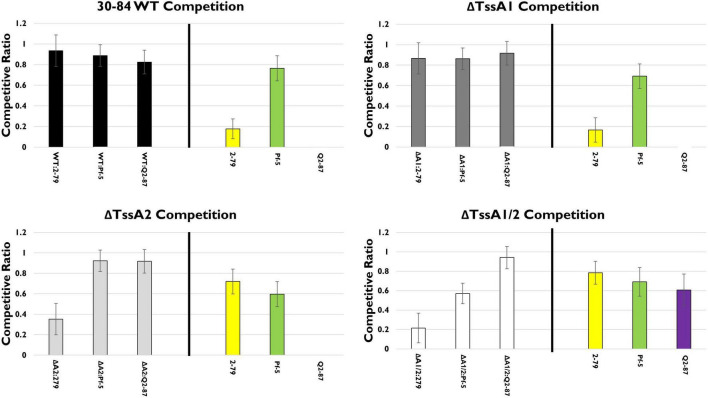
*In vitro* competition assays. The competitive fitness of 30–84 WT, the single T6SS mutants, ΔTssA1 and ΔTssA2, and the double mutant, ΔTssA1/2 were evaluated by comparing their populations when grown separately or in 50:50 mixtures with other *Pseudomonas* rhizosphere colonizing bacteria in liquid media. Data are expressed as competitive ratios (population in mixture/population when grown separately). Gray scale bars (left) indicate performance of 30–84 WT and derivatives, and colored bars (right) indicate performance of competitors, including *P. fluorescens* 2–79 (yellow color, having no T6SS), *P. protegens* Pf-5 (green color, having a T6SS-1 homolog), and *P. fluorescens* Q2–87 (purple color, having three T6SS, including T6SS-1 and T6SS-2 homologs). Individual bacterial cultures or mixture cultures were spotted onto nitrocellulose filters on LB plates and incubated at 28°C, 5 h. Bacterial cells were washed from filters, collected via centrifugation, and populations were enumerated after 48 h via serial dilution plating. Data are the mean competitive ratios of five replicates pooled across three experiments (*n* = 15) and standard errors are indicated.

We explored the hypothesis that disruption of either or both T6SS alters competitive fitness using single strain competition assays *in vitro*. For this assay, 30–84 WT and each of the T6SS mutants were grown separately or in 50:50 mixtures with other well-characterized rhizosphere colonizing *Pseudomonas* PGPB strains shown in this study or previous work to be completely or partially resistant to phenazines or bacteriocins (Dorosky et al., 2017, 2018). These included strains for which there was information about their T6SS and the sequence similarity of their T6SS to the *P. chlororaphis* 30–84 T6SS clusters. Rhizosphere competitors included *P. synxantha* 2–79 (no T6SS), *P. protegens* Pf-5 (a T6SS-1 homolog), and *P. fluorescens* Q2–87 (three T6SS, including T6SS-1 and T6SS-2 homologs) (Loper et al., 2012; Supplementary Figure S1). For mixed strain treatments, strains were grown independently then mixed and applied to filter paper on solid LB medium, whereas for single strain treatments the same total volume of the one strain was applied. The population of each strain in mixture was compared to the populations of their single strain counterparts to observe any effect on growth. Competition with 30–84 WT, ΔTssA1, ΔTssA2, or ΔTssA1/2 are shown as separate analyses (Figure 3). All strains grew well when cultured separately on filters (∼10^8^, and did not differ statistically, *p* < 0.05). In competition with *P. synxantha* 2–79, 30–84 WT and ΔTssA1 reduced *P. synxantha* 2–79 populations, whereas ΔTssA2 and ΔTssA1/2 permitted substantial growth of *P. synxantha* 2–79 in mixed cultures and the growth of both *P. chlororaphis* 30–84 derivatives was reduced. These results suggest T6SS-2 (which may be randomly firing), but not TSS6-1 (which may be contact dependent) confers a competitive advantage to *P. chlororaphis* 30-84 over *P. synxantha* 2-79 (which lacks a T6SS). In contrast, neither of the T6SS had an appreciable effect on the growth of *P. protegens* Pf-5, whereas *P. protegens* was able to reduce the growth ΔTssA1/2 in mixed culture. In competition with *P. fluorescens* Q2–87, 30–84 WT and both single T6SS mutants virtually eliminated *P. fluorescens* Q2–87, whereas ΔTssA1/2 permitted growth of *P. fluorescens* Q2–87 in mixed culture, indicating having at least one T6SS conferred a competitive advantage to 30–84 WT (Figure 3).

**FIGURE 4:**
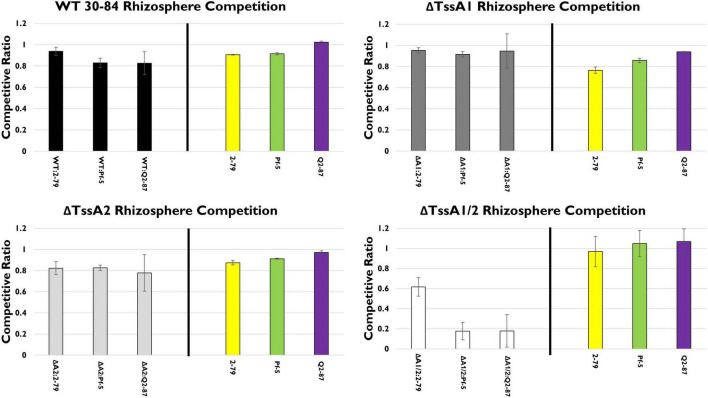
Rhizosphere competition assays. The competitive fitness 30–84 WT, the single T6SS mutants, ΔTssA1 and ΔTssA2, and the double mutant, ΔTssA1/2 were evaluated by comparing their populations when grown separately or in 50:50 mixtures with other *Pseudomonas* rhizosphere colonizing bacteria in the rhizosphere. Data are expressed as competitive ratios (population in mixture/population when grown separately). Gray scale bars (left) indicate performance of 30–84 WT and derivatives, and colored bars (right) indicate performance of competitors, including *P. fluorescens* 2–79 (yellow color, having no T6SS), *P. protegens* Pf-5 (green color, having a T6SS-1 homolog), and *P. fluorescens* Q2–87 (purple color, having three T6SS, including T6SS-1 and T6SS-2 homologs). After 28 days, bacterial populations from the entire root system and loosely adhering soil were collected and enumerated via serial dilution plating. Populations were standardized to root dry weight. Data are the mean competitive ratios of at least eight replicates pooled across three experiments (*n* = 24) and standard errors are indicated.

In the rhizosphere, in mixed treatments with *P. chlororaphis* 30–84 WT or either of the single T6SS mutants and one of the biological control treatments, none of the competitor strains (*P. synxantha* 2–79, *P. protegens* Pf-5, or *P. fluorescens* Q2–87) had a competitive advantage (Figure 4). However, ΔTssA1/2 populations were reduced substantially in the presence of *P. protegens* Pf-5 and *P. fluorescens* Q2–87 and reduced somewhat in the presence of *P. synxantha* 2–79, suggesting that at least one T6SS is needed for competitive fitness in mixtures, especially with the biological control agents having at least one T6SS.

We also examined the importance of one or both T6SS using the wild type and T6SS mutants in competition assays with several environmental isolates or plant pathogens (Supplementary Figure S3). We found that the environmental isolate *P. putida* F1 (no T6SS-encoding genes) performed similarly to *P. synxantha* 2–79 in mixed culture being inhibited by *P. chlororaphis* 30–84 WT and ΔTssA1, whereas ΔTssA2 and ΔTssA1/2 permitted substantial growth. *Agrobacterium tumefaciens* C58 and *Pseudomonas syringae* pv. tomato DC3000, each have functional T6SS (Petnicki-Ocwieja et al., 2002; Ma et al., 2014, respectively) but their populations were reduced substantially when grown with 30–84 WT or either of the single mutants. When grown with the double mutants both of these pathogen competitors were able to maintain high populations. *Pectobacterium carotovorum* subsp. *carotovorum* inhibited the double mutant, but the wild type and the single mutants were able to coexist with the pathogen (Supplementary Figure S3). These results indicate that having at least one T6SS is important for competition against plant pathogens, with T6SS-2 being important for competition against strains lacking their own T6SS.

### T6SS as an Anti-predation Mechanism Against Different Types of Bacterivores

In these studies, we employed three different bacterivores having different feeding styles, including *Dictyostelium discoideum* a soil dwelling amoeba that feeds on bacteria at the soil surface via phagocytosis (Dunn et al., 2018; Williams and Kay, 2018), *Tetrahymena thermophila* a ciliate filter/phagocytosis feeder (Gavin, 1980; Luan et al., 2012; Dürichen et al., 2016), and the soil-dwelling nematode *Caenorhabditis elegans* (Avery and You, 2018). Bacteria used as prey in all feeding assay included 30–84 WT, ΔTssA1, ΔTssA2, ΔTssA1/2, and *P. chlororaphis* derivatives deficient specifically in the production of phenazines (Phz), quorum sensing (QS) signal production, or one or both T6SS system, e.g., 30–84 WT (Phz^+^, QS^+^, T6SS^+^), 30–84 ZN (Phz^–^, QS^+^, T6SS^+^), 30–84 I/I2 (Phz^–^, QS^–^, T6SS^+^), ΔTssA1, ΔTssA2, ΔTssA1/2 (Phz^+^, QS^+^), and 30–84 GacA (Phz^–^, QS^–^, T6SS*^reduced^*).

#### Dictyostelium discoideum

**FIGURE 5:**
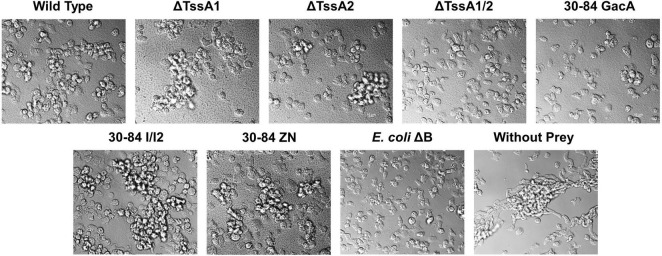
Aggregation behavior of *Dictyostelium discoideum* grown with different bacterial strains as a food source. Bacteria used as prey in the feeding assay included 30–84 WT, ΔTssA1, ΔTssA2, ΔTssA1/2, 30–84 GacA, 30–84 I/I2, or 30–84 ZN and *E. coli*ΔB (used as a preferred prey in the lab). *D. discoideum* without prey bacteria was used as a negative control. *D. discoideum* cells were grown in 24-well plates in low nutrient PBM media for 24 h and aggregation behavior was observed using DIC microscopy (100X oil). The *D. discoideum* control (without prey) showed high levels of aggregation caused by stress due to the lack of nutrition in the media. *D. discoideum* growing with 30–84 WT, ΔTssA1, ΔTssA2, 30–84 I/I2, and 30–84 ZN displayed a similar level of aggregation, indicating that *D. discoideum* cannot eat these strains. *D. discoideum* grown with *E. coli*ΔB showed little to no aggregation. *D. discoideum* growing with ΔTssA1/2 and 30–84 GacA treatments showed similarly low levels of aggregation. Two replicate experiments were performed, and representative images from the same replicate are presented.

To test our hypothesis that one or both of the T6SS function in anti-predation defense against *D. discoideum* amoeba cells, we observed the behavior of *D. discoideum* amoebae when the food source offered as prey consisted of 30–84 WT, 30–84 ZN, 30–84 I/I2 ΔTssA1, ΔTssA2, ΔTssA1/2, or 30–84 GacA. We performed the assay in two different types of medium: low nutrition (PBM) and high nutrition (HL5) medium. Because starvation stress in this amoeba results in aggregation and formation of multicellular fruiting bodies, we assessed aggregation behavior (Kessin, 2001). In low nutrition medium, we observed that after 24 h, *D. discoideum* cells growing without a bacterium food source (control) began to thin and stream and formed extensive aggregates (Figure 5). Similarly, when offered 30-84 WT or derivatives 30–84 ZN, 30–84 I/I2, ΔTssA1, or ΔTssA2, all having at least one intact T6SS, extensive aggregation resulted. In contrast, *D. discoideum* cells growing with *E. coli*ΔB (used in lab as a preferred prey source) showed no aggregation. *D. discoideum* growing with ΔTssA1/2 and 30–84 GacA treatments showed no aggregation, similar to cells grown on *E. coli*ΔB, indicating that the *D. discoideum* was able to gain adequate nutrition by using these strains as a food source (Figure 5). In HL5 media, little to no aggregation behavior was seen, consistent with *D. discoideum* having adequate nutrition (Supplementary Figure S4). These results suggest that having at least one functional T6SS causes *D. discoideum* stress behavior typically associated with starvation in low nutrition media, whereas neither the lack of phenazine or quorum sensing signal production reduced the aggregation behavior, indicating neither play a significant role in promoting the stress response.

**FIGURE 6:**
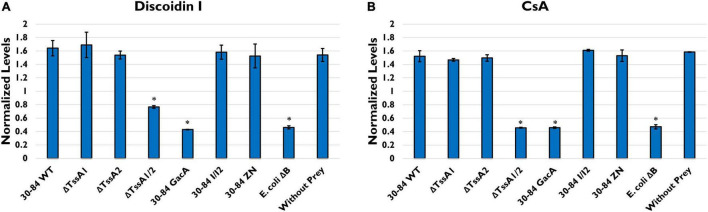
Levels of two starvation markers in *Dictyostelium discoideum*. *D. discoideum* AX2 cells were incubated in PBM with the indicated prey strains (30–84 WT, ΔTssA1, ΔTssA2, ΔTssA1/2, 30–84 GacA, 30–84 I/I2, or 30–84 ZN and *E. coli* ΔB) or without prey (negative control) for 24 h. Western blots were stained with anti-Discoidin I, anti-CsA, or anti-beta-Actin antibodies. Graphs show the levels of **(A)** Discoidin I or **(B)** CsA normalized to total actin. Values are mean and standard error of 2 independent experiments. * Indicates *p* < 0.01 compared to *D. dictyostelium* AX2 control (Unpaired *t*-tests, Welch’s correction).

To confirm that bacterial T6SS induce stress during feeding, we measured the production of *D. discoideum* development markers Discoidin I and Contact site A (CsA). Discoidin I is a protein involved in adhesion that is detectable at low levels in vegetative amoeba cells but is expressed at high levels during aggregation (Springer et al., 1984). CsA is a glycoprotein involved in cell-cell binding that is expressed at very low levels in growing cells and is then expressed during development (Harloff et al., 1989). We found that when grown without prey (negative control) in low nutrition media, *D. discoideum* produced high levels of both Discoidin I and CsA (Figure 6). When grown with *E. coli* ΔB (preferred prey) the production of both proteins was significantly lower compared to the negative control. When grown with 30–84 WT, 30–84 I/I2, or 30–84 ZN as the prey, levels of Discoidin I and CsA were comparable to the negative control, indicating that phenazines were not the driving mechanism behind this response. *D. discoideum* also produced high levels of both development markers when grown with ΔTssA1 and ΔTssA2. When *D. discoideum* was grown with ΔTssA1/2 or 30–84 GacA, levels of both proteins were comparable to the *E. coli* ΔB control (Figure 6). Taken together these results indicate that bacteria having one or both T6SS causes stress in *D. discoideum*, which deters predation.

**FIGURE 7:**
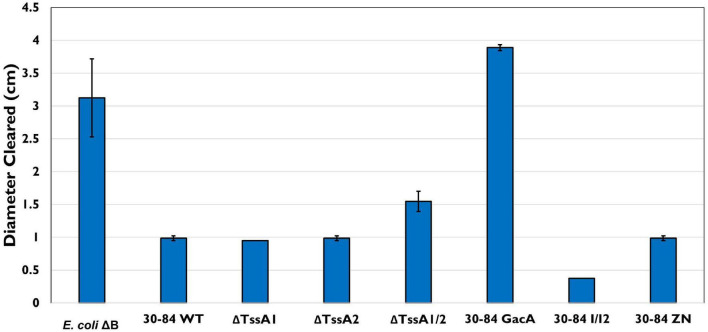
Bacterial plate clearing by *Dictyostelium discoideum.* Bacteria used as prey in the feeding assay included 30–84 WT, ΔTssA1, ΔTssA2, ΔTssA1/2, 30–84 GacA, 30–84 I/I2, or 30–84 ZN and *E. coli*ΔB (used as a preferred prey in the lab). The diameter of bacterial lawn cleared by *D. discoideum* was measured in cm after 72 h. Lettering indicates significant differences. *D. discoideum* colonies grown on 30–84 WT, both single mutants, 30–84 I/I2, and 30–84 ZN showed little to no clearing, indicating low to no bacterial cell death. *D. discoideum* growing on *E. coli*ΔB showed significant levels of clearing. When grown on 30–84 GacA, levels of clearing similar to the control were observed. When grown on ΔTssA1/2, less clearing was observed than on the control, but levels were still significantly higher than clearing on 30–84 WT and the single mutants, indicating a decrease in bacterial fitness when lacking at least one functional T6SS. Data are the means and standard error (may be too small to see for some treatments) of four biological replicate experiments (*n* = 4). Data were analyzed using one-way ANOVA and Student *t*-tests, *p* < 0.05.

To determine how *D. discoideum* predation effects bacterial fitness, a plate clearing assay was performed in which the size of the zone of clearing (and *D. discoideum* spread) after 5 days was used as a measure of bacterial cell death due to predation. On all plates, the initial colony of *D. discoideum* resulted in a clear zone ∼1 cm in diameter after 2 days. After 5 days, the clearing zone on plates containing 30–84 WT, ΔTssA1, ΔTssA2, 30–84 I/I2, and 30–84 ZN did not increase significantly i.e., the zones were within 0.05 cm of the clearing diameter measured at day 2 (Figure 7). The clearing zone on plates containing the preferred prey *E. coli*ΔB or 30–84 GacA grew to 3.0 ± 0.2 and 3.8 ± 0.02 cm, respectively, and on ΔTssA1/2 grew to 1.7 ± 0.06 cm. These results indicate that 30–84 GacA and ΔTssA1/2 populations suffered greater losses due to *D. discoideum* feeding (Figure 7).

#### Tetrahymena thermophila

**FIGURE 8:**
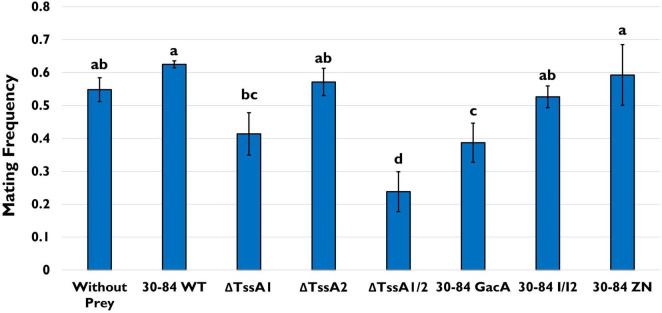
*Tetrahymena* mating assay. *T. thermophila* CU427 and CU330 were grown separately overnight in Tris Buffer (pH = 7.4) to induce starvation. Populations were standardized to a cell density of 1.5 – 2 × 10^5^/10 mL (via direct counts) in fresh Tris Buffer and mixed together with only the bacterial strain as a food source. Bacteria used as prey included 30–84 WT, ΔTssA1, ΔTssA2, ΔTssA1/2, 30–84 GacA, 30–84 I/I2, or 30–84 ZN, and the *T. thermophila* pairs without prey was used as a negative control. After 4 h, the treatments were viewed using a Leitz (Epivert) microscope (100X magnification), and the frequency of mated cell pairs (number of mated pairs/total number of observations) determined. Mean frequencies and standard errors are shown. This experiment was repeated three times (*n* = 3). Letters denote significant differences. Data were analyzed using one-way ANOVA and Student *t*-tests, *p* < 0.05.

As above, in the feeding assay using the model ciliate *Tetrahymena thermophila* CU427, predators were offered the same single prey choice strains (30–84 WT, ΔTssA1, ΔTssA2, ΔTssA1/2, 30–84 GacA, 30–84 I/I2, 30–84 ZN), and population densities of the predator in mixed culture were measured after 4 and 24 h. No significant differences in *T. thermophila* population densities were found even after 24 h (Supplementary Figure S5). However, we also assessed stress related behaviors of *T. thermophila* when offered the different prey choices via a mating assay. Two *T. thermophila* of different mating types, CU427 and CU330, were selected for this assay as they are recognized as “non-self” cells with different germlines, and will therefore reproduce (Cervantes et al., 2013). In the mating assay, *T. thermophila* were starved prior to their exposure to the different prey. If *T. thermophila* continued to experience starvation stress, this would increase instances of mating (Cole, 2000-2013). After 4 h, the frequency of mating was ∼55% for the control (no food source) and the frequency of mating for 30–84 WT, 30–84 I/I2, or 30–84 ZN as the food source was similar (62, 53, and 62%, respectively; Figure 8). Since all three of these derivatives have wild type T6SS expression, but differ in their ability to produce phenazines, these data demonstrate that the production of phenazines did not enhance mating. In contrast, the frequency of mating was significantly lower for *T. thermophila* growing with ΔTssA1/2 or 30-84 GacA (19%, 36%, respectively, Figure 8). Observations support the hypothesis that having an intact T6SS causes predator stress, resulting in reduced levels of bacterivory.

#### Caenorhabditis elegans

As in the previous experiments, the bacterivorous nematode *C. elegans* was offered different prey choices. In this experiment, 5 *C. elegans* adults were allowed to graze for 1 h on a lawn of each bacterial strain and then moved successively to a fresh prey-containing plate every 1 h three more times, enabling nematodes to lay eggs on each of the plates. Plates were then observed for 72 h at 24 h intervals and adult and juvenile nematodes maturing from eggs were counted. Well-fed nematodes will mature from eggs within 72 h.

**FIGURE 9:**
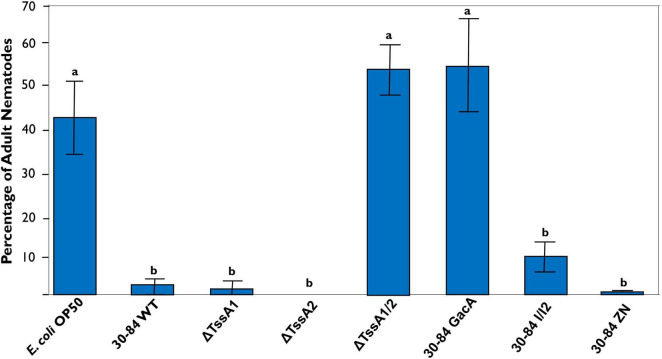
Percentage of *C. elegans* that reach maturity after 72 h. Five adult *C. elegans* were transferred at 1-h intervals to new prey-containing plates to facilitate egg laying (a total of four successive transfers), and then the percentage of nematodes maturing to adults were measured every 24 h over a 72-h period. Plates contained either 30–84 WT, ΔTssA1, ΔTssA2, ΔTssA1/2, 30–84 GacA, 30–84 I/I2, 30–84 ZN, or *E. coli* OP50 (control) as a food source. Data are the mean and standard errors of three replicate experiments (four plates/replicate). Data were analyzed using one-way ANOVA and Tukey tests. Letters indicate significant differences, *p* < 0.01.

After 72 h, a large percentage (∼45%) of nematodes grown on *E. coli* OP50, the *C. elegans* normal laboratory food source, were mature (Figure 9). The percentages of adult nematodes observed on plates having ΔTssA1/2 and 30–84 GacA as food sources were also high (∼55%), whereas the percentages were lower when *C. elegans* was grown on plates having 30–84 WT, ΔTssA1, ΔTssA2, 30–84 I/I2, or 30-84 ZN as the food source (2, 2, 0, 9.5, and 0.03% adults, respectively) (Figure 9). Moreover, for plates containing 30–84 WT, 30–84 I/I2, or 30–84 ZN as the food source, *C. elegans* avoided the center of the plates where the bacterial density was greatest, instead moving to the edges of the plate (Supplementary Figure S6). On plates containing these prey sources, less than 25% of the plate was cleared (where clearing indicates bacterial consumption, Supplementary Table S2). Since these three derivatives have wild type T6SS expression, but differ in their production of phenazines, these data demonstrate that the production of phenazines was not the primary feeding deterrent. Similarly, the nematodes moved to the outside of the plate and generally cleared less than 25% of the plate when grown on plates containing ΔTssA1 or ΔTssA2 as the food source. On ΔTssA1/2 and 30–84 GacA, nematodes were found in the center of the plates and often cleared greater than 90% of the plate (Supplementary Figure S6 and Supplementary Table S2). Data indicate that having either T6SS is a deterrent to *C. elegans* feeding, and that *C. elegans* prefer prey lacking expression of both.

### *In silico* Identification of Putative T6SS-Dependent Effectors and Immunity Proteins

Given the importance of both systems for defense against competitors and predators, we used a bioinformatics approach to identify potential T6SS effectors and their predicted modes of action that may contribute to defense. Neither of the T6SS clusters in *P. chlororaphis* 30–84 contain genes previously annotated as encoding T6SS-dependent effectors. However, since T6SS-dependent effector genes are often located in close proximity to genes encoding VgrG, Hcp, or PAAR proteins, we analyzed the predicted protein sequences of genes closely associated with all copies of these genes using BLASTp (Lien and Lai, 2017; Spiewak et al., 2019). Putative effectors and immunity proteins were identified based on predicted functional domains and homology to proteins within established T6SS effector superfamilies. Using this approach, a diversity of putative T6SS effectors and associated proteins were discovered and all VgrG- or Hcp-encoding genes, whether located within a T6SS gene cluster or elsewhere in the genome, were associated with at least one putative effector (Supplementary Table S3).

Not surprisingly the two T6SS gene clusters harbored genes encoding different putative effectors. Within the T6SS-1 cluster, adjacent to an Hcp encoding gene is a gene encoding a Tae4-like protein (RS17725). Tae (type VI amidase effectors) family effectors have peptidoglycan amidase activity and are typically located adjacent to a cognate immunity protein (Zhang et al., 2013). However, the adjacent gene encoding a hypothetical protein bears no amino acid sequence homology to the known Tae4 immunity protein Tai4 and has no well characterized conserved domains. Also within the T6SS-1 cluster and adjacent to a VgrG-encoding gene (RS17755) are two genes encoding hypothetical proteins, one with a DUF2169 domains. Adapter proteins with the DUF2169 domain were shown to be necessary for the efficient secretion of T6SS effectors in *A. tumefaciens* (Bondage et al., 2016). Located nearby the T6SS-1 gene cluster and associated with a VgrG-encoding gene (RS17845) are genes encoding three hypothetical proteins predicted to have lipase (class 3) activity, be a cytoplasm-localized lipoprotein, or contain a DUF4123 domain. Proteins having a DUF4123 domain may serve as T6SS effector chaperones (Liang et al., 2015). In the T6SS-2 cluster, a gene encoding a hypothetical protein (RS29475) having a lysozyme-like domain similar to the C-terminal domain of pesticin is flanked by two VgrG encoding genes. Pesticin is an antibacterial toxin involved in hydrolysis of the peptidoglycan in the periplasm. Proteins with a pesticin C-terminal domain are typically involved in the hydrolysis of beta-1,4- linked polysaccharides (Patzer et al., 2012). Also in the T6SS-2 cluster and adjacent to one of the VgrG encoding genes are genes encoding three hypothetical proteins, one with a DUF4123 domain, one encoding a Tli1-like immunity protein with a DUF3304 domain shown to be associated with immunity proteins (Crisan et al., 2019), and one encoding a Tle1-like phospholipase protein (Ma et al., 2017) with a DUF2235 domain shown to be common in proteins containing a MIX (marker for type six effectors) sequence (Salomon et al., 2014).

Elsewhere in the genome, we discovered genes encoding putative effectors with predicted activities related to the breakdown of phospholipids or the production of toxins. Including the Tle-like effector associated with T6SS-2, we discovered three putative effectors predicted to have phospholipase activity, belonging to different phospholipase effector families: Tle, Tpl, and Pld. A gene encoding a TplE-like protein (RS20065) is located in a cluster with genes encoding a VgrG, two putative immunity protein pairs (paralogs), and a PAAR protein. A gene encoding a putative type VI secretion phospholipase D effector (Pld) in the same ortholog group as *tle5B* in *P. aeruginosa* PAO1 (RS20580) was located adjacent to another VgrG encoding gene. In *P. aeruginosa* PAO1, adjacent to *tle5B* are genes encoding three immunity proteins, each were found to have four, three, or two Sel1-like repeats (SLR) (Wen et al., 2020). No genes encoding similar immunity proteins were found in *P. chlororaphis* 30–84, however, the gene adjacent to *pld* in *P. chlororaphis* 30–84 encodes a hypothetical protein with three SLR repeats. Elsewhere in the genome, a gene encoding a RhsA- like protein (RS01885) was found within a four gene cluster containing genes encoding a putative TAP (T6 adaptor protein) protein with unknown function, VgrG and Hcp. Rhs (rearrangement hotspot) proteins, typically have multiple repeats of RHS sequences and have been associated with toxicity and bacterial competition (Koskiniemi et al., 2013; Vacheron et al., 2019). The arrangement of the four genes is the same as the RhsP2-containing cluster in *P. aeruginosa* PA14 previously shown to be involved in T6SS secretion (Jones et al., 2014). Other putative effectors and their predicted function are also described in Supplementary Table S3.

## Discussion

Type VI Secretion Systems (T6SS) are known to be involved in many different types of bacterial interactions with prokaryotes and eukaryotes, and previous studies showed that in comparison to bacterial strains lacking T6SS, the presence of a T6SS confers greater fitness to bacteria in their environments (Haapalainen et al., 2012; Bernal et al., 2017b). Many bacterial species possess one to several T6SS and the different T6SS can be involved in different types of interactions (Chen et al., 2011). Consequently, possessing more than one T6SS can increase the repertoire of potential benefits to the strain (Schwarz et al., 2010). Plant growth promoting pseudomonads may either lack T6SS or possess one to several different T6SS, but their importance in activities related to their plant-associated habitat are not well studied. In the present study, we describe the structure and organization of two separate T6SS gene clusters in the PGPB strain *P. chlororaphis* 30–84 and provide evidence to support a role for both in competition against other bacterial species, including other PGPB and phytopathogens, and protection against different types of bacterivorous predators.

Bioinformatic analysis revealed that *P. chlororaphis* 30–84 has two separate T6SS clusters, each of which contain at least 12 of the 13 conserved T6SS structural proteins, but that the two clusters are quite different in terms of their organization and regulation. Comparative genomic analyses suggested that the two systems are not redundant and differ in their potential for responding to environmental stimuli. The T6SS-1 gene cluster contains putative Tag protein-encoding genes, which are involved in contact dependent firing, and was organizationally similar to the gene cluster encoding the *P. aeruginosa* T6SS previously shown to display dueling behavior (Basler et al., 2013). Together, these findings led us to hypothesize that T6SS-1 is fired in a contact dependent manner. The absence of the Tag-encoding genes in the T6SS-2 cluster led us to hypothesize random firing of this T6SS (i.e., not contact-dependent). Consistent with these hypotheses, *P. chlororaphis* 30-84 derivatives lacking a functional T6SS-2 (ΔTssA2 and ΔTssA1/2), and thus lacking random firing, permitted substantial growth of *P. synxantha* 2–79 (which lacks a T6SS) in mixed cultures, whereas derivatives having the T6SS-2 cluster (30–84 WT and ΔTssA1) competitively reduced *P. synxantha* 2–79 *in vitro*. We saw the same pattern of behavior in competition assays using *P. putida* F1, which also lacks a T6SS.

Previous studies showed that T6SS are important for the inhibition of competitors. For example, Bernal et al. (2017a) showed the biological control strain *P. putida* KT2440 possesses three different T6SS and, when all three were disrupted, control of certain plant pathogens was lost. In the present study, we examined competitive fitness both in terms of the ability of the *P. chlororaphis* mutants to limit the growth of challengers and to resist the impact of challenges on their own growth. We found that *P. chlororaphis* wild type was significantly more effective than the double mutant in inhibiting the growth of several different plant pathogens and even different rhizosphere-colonizing biological control strains in mixed cultures *in vitro*. Moreover, in competition with certain strains (*P. synxantha* 2–79, *P. protegens* Pf-5, *Pectobacterium carotovorum*), the population of the double mutant was reduced, indicating inability to protect itself. Competition assays utilizing the single mutants showed that, in most cases, having either system was sufficient for competitor inhibition, although as noted above there were some exceptions demonstrating that T6SS-2 was effective against a broader spectrum of competitors (including those having no T6SS) than T6SS-1. It was interesting that derivatives having either T6SS system significantly inhibited *P. fluorescence* Q2–87 (having three T6SS, including T6SS-1 and T6SS-2 homologs) in mixed cultures *in vitro*, whereas *P. fluorescence* Q2–87 was able to grow in the presence of the double mutant. These observations are consistent with the hypothesis that despite having multiple T6SS systems, Q2–87 may not have immunity to *P. chlororaphis* 30–84 T6SS effectors. In some cases, pathogens (*Pseudomonas marginalis*) or biological control agents (*Pseudomonas putida* KT2440) that were chosen for competition assays were highly sensitive to phenazine or bacteriocin production and would not grow in the competition assay even with ΔTssA1/2. In other cases, strains (*P. putida* F1, *P. syringae* DC3000) that were shown previously to be sensitive to bacteriocins produced by *P. chlororaphis* 30–84 (Dorosky et al., 2017) were able to sustain somewhat higher populations in mixed culture with the double mutant compared to wild type, indicating both types of weapons contribute to competitive fitness against certain *Pseudomonas* strains. However, the majority of biological control strains tested (*P. synxantha* 2–79, *P. protegens* Pf-5, *P. fluorescens* Q2–87) were neither sensitive to phenazines nor bacteriocins, highlighting the importance of contact-dependent mechanisms in a rhizosphere where the antibiotic “resistome,” the collection of all the antibiotic resistance genes, may be well established (O’Brien and Wright, 2011).

Interestingly, the rhizosphere habitat offered some protection from competition, presumably because strains could escape interaction spatially. In general, ΔTssA1/2 was less competitive than wild type. This deficit is unlikely to be the result of insufficiency in colonization ability, because ΔTssA1/2 colonized the rhizosphere as well as 30–84 WT in sterile and field soil in our persistence assay. However, after five harvest cycles ΔTssA1/2 populations were significantly smaller than 30–84 WT populations, but only in field soil. The populations of ΔTssA2 were also reduced under these conditions, suggesting that the putatively random-firing T6SS-2 is slightly more important than the putatively contact-dependent firing T6SS-1 for *P. chlororaphis* 30–84 competitive persistence in the rhizosphere. In this study, we purposely looked at competition between *P. chlororaphis* 30–84 and other well-characterized PGPB strains because these are also known to be good rhizosphere colonists. Our results highlight the importance of considering T6SS compatibility when considering multi-strain mixtures of PGPB.

We also examined the importance of T6SS for protecting *P. chlororaphis* 30–84 from predators with different feeding styles including, *D. discoideum, T. thermophila*, and *C. elegans.* Previous studies using these organisms as host model systems demonstrated that bacterial T6SS play a vital role in virulence against *D. discoideum* and *C. elegans* and protection against predation by *Tetrahymena* (Pukatzki et al., 2006; Sana et al., 2013; Wang et al., 2018). Our study is unique in that we examined the effect of each T6SS on the feeding and behavior of the predators and the impact of predation on bacterial populations rather than focusing on virulence and killing. For all three predators, we found that being grown with a bacterial food source with least one T6SS reduced predator feeding, increased predator stress, and altered predator behavior relative to the double mutant. For example, when grown with 30–84 WT or the single mutants as the primary food source, *D. discoideum* formed extensive aggregates, a common stress response (Kessin, 2001). Levels of developmental proteins related to aggregation behavior (Discoidin I and CsA) were also high in *D. discoideum* populations grown on 30–84 WT or the single mutants as the primary food source. In contrast, when grown with the double mutant or 30–84 GacA as the primary food source, no aggregation was observed, and development protein levels were reduced. When grown together with the different prey strains in high nutritional medium, there were no differences in predator behavior or stress levels, indicating no limitations to co-existence when there was sufficient food for the predator. In assays using pre-starved *T. thermophila*, mating, a stress related behavior (Cole, 2000-2013), was observed at high levels only when *T. thermophila* was grown with 30–84 WT or the single mutants as the primary food source. *C. elegans* also displayed behavior indicating its avoidance of 30-84 WT or single mutants, moving away from where the bacteria were spotted to the edges of the plates. In contrast, when grown with the double T6SS mutant or 30–84 GacA as the primary food source, nematodes were found in the center of the plates and matured at higher rates. Having at least one T6SS also had important consequences for bacterial fitness, resulting in less herbivory of those derivatives with at least one T6SS than for those without a functional T6SS as determined from clearing zones in assays with *D. discoideum* and *C. elegans*.

Phenotypic variation resulting from spontaneous mutation in *gacS/gacA* is a common problem among *Pseudomonas* biological control agents (van den Broek et al., 2005). Spontaneous *gacS* or *gacA* mutants arising in fermentation culture may outcompete the antibiotic producing wild type, causing a deficiency in secondary metabolites essential for biological control activity (Duffy and Defago, 2000). However, in the rhizosphere *P. chlororaphis* 30–84 wild type and *gac* mutants survive better together than apart (Chancey et al., 2002) and interact mutualistically in biofilms (Driscoll et al., 2011), indicating a benefit to maintaining phenotypic variation for competitive rhizosphere fitness. As in our study, previous work by Jousset et al., 2009 demonstrated that *C. elegans* and the amoeba, *Acanthamoeba castellanii*, preferentially consumed a *Pseudomonas protegens* (previously *fluorescens*) CHA0 *gacS* mutant – although in their study they used mixed populations of mutants with wild type. Jousset et al. (2009) suggested that predator preference for the *gacS* mutant was due to loss of quorum sensing and antibiotic production. Like *P. chlororaphis* 30–84, *P. protegens* CHA0 possesses a diverse arsenal of diffusible weapons as well T6SS-encoding genes. In the present study, having a collection of phenazine, quorum sensing, and T6SS mutants enabled us to demonstrate that the T6SS had an important role in altering predator feeding and behavior. However as determined from the plate clearing assays with *D. discoideum* and *C. elegans*, *P. chlororaphis* 30–84 GacA was somewhat more aggressively attacked than ΔTssA1/2, indicating that other GacS/GacA controlled phenotypes contribute to defense against these predators.

Previous work demonstrated a role for T6SS in the delivery of effectors important for competition and virulence (Russell et al., 2011; Chen et al., 2015; Bernal et al., 2017b). All effectors delivered by T6SS studied to date are associated with the needle-like structure, specifically with either VgrG, Hcp or PAAR proteins (Ho et al., 2014). We identified a number of potential effector-encoding genes in the *P. chlororaphis* 30–84 genome that could be delivered via T6SS based on their proximity to genes encoding VgrG, Hcp or PAAR proteins, a method commonly used for effector discovery (English et al., 2012; Spiewak et al., 2019). Notably the putative effectors identified included several genes that may encode proteins with lipase or phospholipase activity, although the functionality of these was not specifically addressed in this study. Previous studies showed that predicted lipases are often encoded adjacent to *vgrG* homologs and those associated with T6SS may function to degrade bacterial membrane phospholipids (Russell et al., 2013). T6SS secreted phospholipase effectors were shown to increase virulence against amoebae (Pukatzki et al., 2006; MacIntyre et al., 2010), and some studies have attributed this to effectors specifically targeting lipids in the membrane of eukaryotes (Miyata et al., 2011; Durand et al., 2014; Jiang et al., 2014). The presence of potential phospholipase effectors in *P. chlororaphis* 30–84 suggests a potential mechanism for the interaction observed with eukaryotic bacterivores. Furthermore, the gene encoding the putative Pld effector is adjacent to a gene encoding a protein with multiple Sel1-like repeats (SLR). Sel1, first described in *C. elegans*, and proteins with SLRs were shown to be important for signal transduction in both prokaryotes and eukaryotes (Mittl and Schneider-Brachert, 2007). An effector with multiple SLRs could indicate another potential mechanism by which *P. chlororaphis* 30–84 effectors interact with eukaryotic predators. In addition, we also found possible effectors with potential amidase, lysozyme, and peptidoglycan degrading activity as well as an Rhsp2 homolog. Rhs effectors were shown to have antibacterial properties and are involved in bacterial competition (Hachani et al., 2014; Whitney et al., 2014; Alcoforado Diniz and Coulthurst, 2015). Notably, adjacent to genes encoding VgrG genes we often found genes encoding proteins with DUF4123 or DUF2169 domains. These domains were shown to be utilized in effector chaperoning (Liang et al., 2015) or secretion (Bondage et al., 2016), suggesting they may be important for cargo loading and secretion in *P. chlororaphis* 30-84 T6SS. Although demonstrating the efficacy of potential effectors was outside the scope of this study, their association with genes encoding VgrG and proteins with predicted chaperone domains strongly indicates their potential role as effectors. The diversity of effectors found and their predicted activities, portrays likely mechanisms for targeting both prokaryotic and eukaryotic systems.

In summary, our results demonstrate that in *P. chlororaphis* 30–84 both T6SS-1 and T6SS-2 serve as defensive weapons against other rhizosphere colonists that may be completely or partially resistant to the production of diffusible antimicrobials and bacteriocins, with T6SS-2 being more important for competition against strains lacking their own T6SS. Having at least one T6SS was also necessary for predator defense whereas loss of phenazine production had little effect on predation or predator behavior, indicating a greater role for T6SS than phenazines in defense against the predators we studied. Interestingly, having only one functional T6SS typically provided the same level and spectrum of protection as wild type. One possible explanation is that both T6SS systems may be capable of interchangeably delivering the repertoire of effectors, although this remains to be tested. Consequently, it is noteworthy that most of the groups of *vgrG*-associated potential effector genes are widely distributed throughout the genome rather than being localized within T6SS gene clusters, and typically one of the associated proteins encoded within each group shares a conserved domain previously shown to serve as a T6SS effector chaperone. We speculate that in contrast to diffusible weapons that may not be produced at low cell density or that function only after attaining a sufficient concentration in the environment, T6SS afford rhizosphere colonizing bacteria an additional more immediate line of defense against competitors and predators.

## Data Availability Statement

The original contributions presented in the study are included in the article/Supplementary Material, further inquiries can be directed to the corresponding author/s.

## Author Contributions

EB, EP, and SK designed the experiments. EB and SK performed the experiments, generated original data, and performed the data analysis. EB and EP wrote the manuscript. EP provided project supervision and major contribution to the final draft. All authors contributed to the interpretation of results and revisions of the manuscript, read and approved the submitted version.

## Conflict of Interest

The authors declare that the research was conducted in the absence of any commercial or financial relationships that could be construed as a potential conflict of interest.

## Publisher’s Note

All claims expressed in this article are solely those of the authors and do not necessarily represent those of their affiliated organizations, or those of the publisher, the editors and the reviewers. Any product that may be evaluated in this article, or claim that may be made by its manufacturer, is not guaranteed or endorsed by the publisher.

## Supplementary Material

Supplementary Figure S1 Phylogenetic tree comparing TssB from *P. chlororaphis* T6SS-1 and T6SS-2 clusters to known homologs. The amino acid sequences for TssB from both *P. chlororaphis* 30–84 T6SS clusters were compared to the corresponding sequences from individuals within each established clade referenced in Bernal et al. (2017b) using NCBI BLASTp. The *P. chlororaphis* 30–84 amino acid sequences and representative TssB amino acid sequences from these clades were aligned and a maximum likelihood tree was constructed using MEGA7. The program FigTree was used to convert the MEGA7 tree into figure format. TssB in the *P. chlororaphis* 30–84 cluster 1 (indicated by stars) aligned with sequences in clade 3 whereas the TssB from *P. chlororaphis* 30-84 cluster 2 (indicated by triangles) aligned predominately with sequences in clade 1.1 (although a single strain belonging to 4A clustered with it).

Supplementary Figure S2 Planktonic growth curve and attached biofilm production. **(A)** Planktonic growth: bacteria were grown in LB medium at 28°C with agitation and populations were measured (OD_620_) every hour up to 8 h and then every 2 h from 24 h to 30 hours. Data are the mean and standard error of six replicates. **(B)** Attached biofilms: separate wells of 96-well plates were inoculated with bacteria and grown at 28°C without agitation for 72 h. After removal of non-adhering cells, surface-attached biofilms were stained with crystal violet. The optical density (540 nm) of crystal violet released from the biofilms was used as a relative measure of biofilm population density. Data are the means and standard errors of two biological replicates per strain (started from separate colonies) and five technical replicates performed three times. Strains tested included 30–84 WT, the single T6SS mutants ΔTssA1 and ΔTssA2, and the double mutant ΔTssA1/2. Data were analyzed using a one-way ANOVA and Tukey’s tests, *p* < 0.05. No significant differences were found.

Supplementary Figure S3 *In vitro* competition assays against environmental isolates and pathogens. The competitive fitness of 30–84 WT and T6SS mutants against environmental isolates and pathogenic strains were evaluated by comparing their populations when grown separately or in 50:50 mixtures in liquid medium. Data are expressed as competitive ratios (population in mixture/population when grown separately). Gray scale bars (left) indicate performance of 30–84WT and derivatives, and colored bars (right) indicate performance of competitors, including *P. putida* F1 (blue), *Agrobacterium tumefaciens* C58 (orange), *P. syringae* DC3000 (red), and *Pectobacterium carotovorum* (green). Individual bacterial cultures or mixed cultures were spotted onto nitrocellulose filters on LB plates and incubated at 28°C, 5 h. Bacterial cells were washed from filters, collected via centrifugation, and populations were enumerated after 48 h via serial dilution plating. Data are the means (log10 CFU/1 mL) of at least five biological replicates/treatment pooled across at least two experiments and standard errors are indicated.

Supplementary Figure S4 Aggregation behavior of *Dictyostelium discoideum* grown with different bacterial strains in high nutrition HL5 medium. Bacteria used as prey in the feeding assay included 30–84 WT, ΔTssA1, ΔTssA2, ΔTssA1/2, 30–84 GacA, 30–84 I/I2, or 30–84 ZN and *E. coli*ΔB (used as a preferred prey in the lab). *D. discoideum* without prey bacteria was used as a negative control. *D. discoideum* cells were grown in 24 well plates in high nutrient HL5 media for 24 h and aggregation behavior was observed using DIC microscopy (100X oil). All treatments showed no aggregation behavior, indicating no stress. Two replicate experiments were performed, and representative images are presented from the same replicate are presented.

Supplementary Figure S5 *Tetrahymena* populations after 24 h feeding assay. Bacteria used as prey in the feeding assay included 30–84 WT, ΔTssA1, ΔTssA2, ΔTssA1/2, 30–84 GacA, 30–84 I/I2, or 30–84 ZN. *T. thermophila* without prey bacteria was used as a negative control. Bacterial and *T. thermophila* cultures were mixed (5 mL, 20 mL, respectively) and grown in 50 ml tubes with agitation (200 rpm, 27°C). After 24 h, *T. thermophila* populations in mixed cultures were enumerated via direct counts using a hemocytometer. Data are the means and standard errors of six replicate experiments. Data were analyzed using a one-way ANOVA and Student *t*-tests (*p* < 0.05). No significant differences were found.

Supplementary Figure S6 Images of *C. elegans* on plates containing different prey after 72 h. Five adult *C. elegans* were transferred at 1-hour intervals to new prey-containing plates to facilitate egg laying (a total of four successive transfers), and then images were taken at 72 h. Images are of the center of the plate where bacterial cultures were applied. Plates contained either 30–84 WT, ΔTssA1, ΔTssA2, ΔTssA1/2, 30–84 GacA, 30–84 I/I2, 30–84 ZN, or *E. coli* OP50 (control) as a food source. Images were obtained using a Zeiss Stemi SV11 scope (26X magnification) and a Hamamatsu ImagEM EM-CCD camera. Adult nematodes can be seen on the plates containing ΔTssA1/2, 30-84 GacA or *E. coli* OP50 (ΔTssA1/2 image contains air bubble), whereas no or only a few, immature nematodes can be seen on plates with the other prey sources. The experiment and imaging were repeated three times with four plates/replicate (*n* = 12) and representative images from the same replicate are shown.
