# Supplementary figures and images for: The Type VI Secretion Systems in Plant-Beneficial Bacteria Modulate Prokaryotic and Eukaryotic Interactions in the Rhizosphere
Source: Front Microbiol. 2022 Apr 7;13:843092. doi: 10.3389/fmicb.2022.843092 (PMC9022076; doi:10.3389/fmicb.2022.843092)

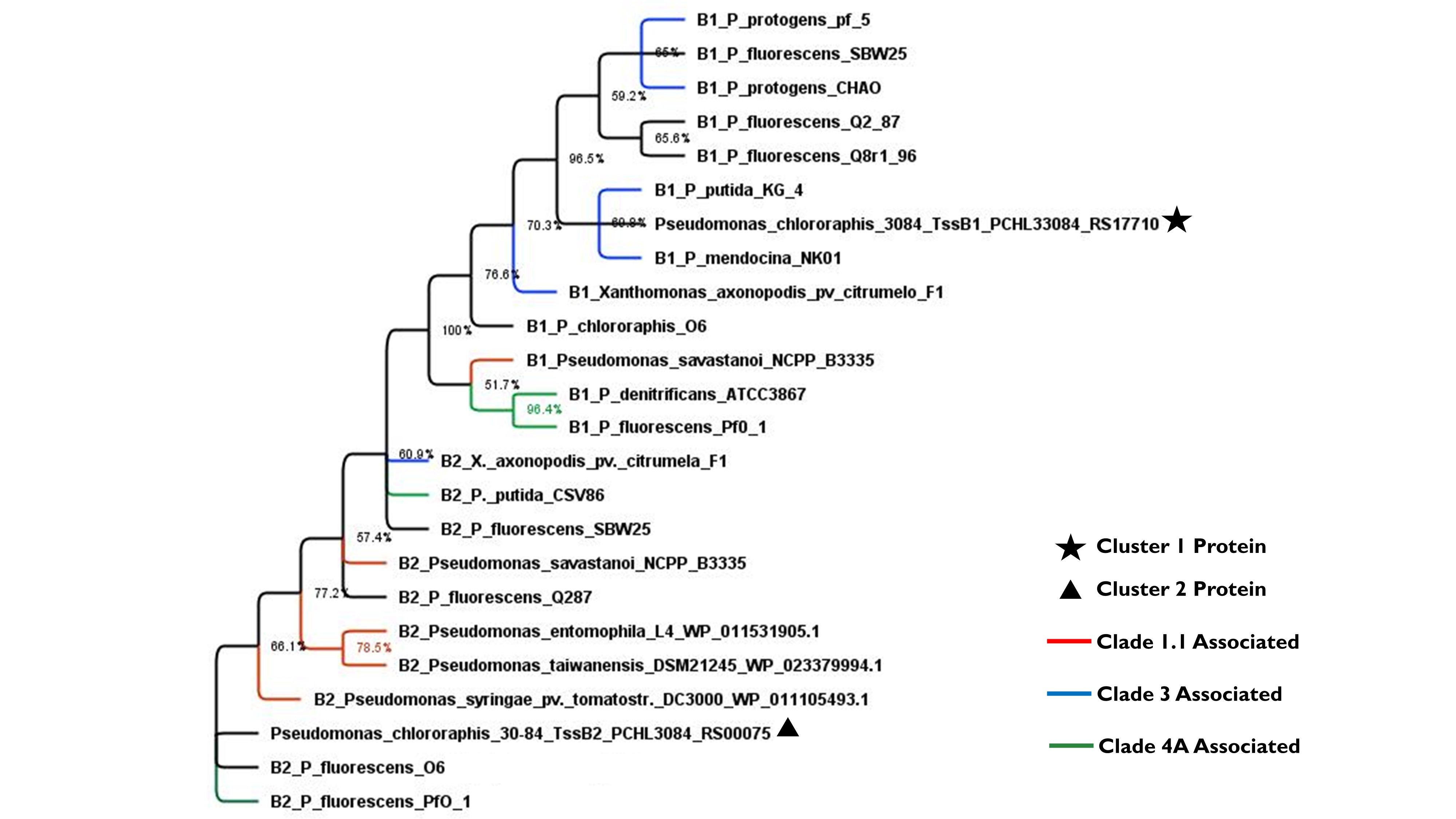

Supplement: Supplementary Figure S1 — Phylogenetic tree comparing TssB from P. chlororaphis T6SS-1 and T6SS-2 clusters to known homologs. The amino acid sequences for TssB from both P. chlororaphis 30–84 T6SS clusters were compared to the corresponding sequences from individuals within each established clade referenced in Bernal et al. (2017b) using NCBI BLASTp. The P. chlororaphis 30–84 amino acid sequences and representative TssB amino acid sequences from these clades were aligned and a maximum likelihood tree was constructed using MEGA7. The program FigTree was used to convert the MEGA7 tree into figure format. TssB in the P. chlororaphis 30–84 cluster 1 (indicated by stars) aligned with sequences in clade 3 whereas the TssB from P. chlororaphis 30-84 cluster 2 (indicated by triangles) aligned predominately with sequences in clade 1.1 (although a single strain belonging to 4A clustered with it). [file Image_1.JPEG]

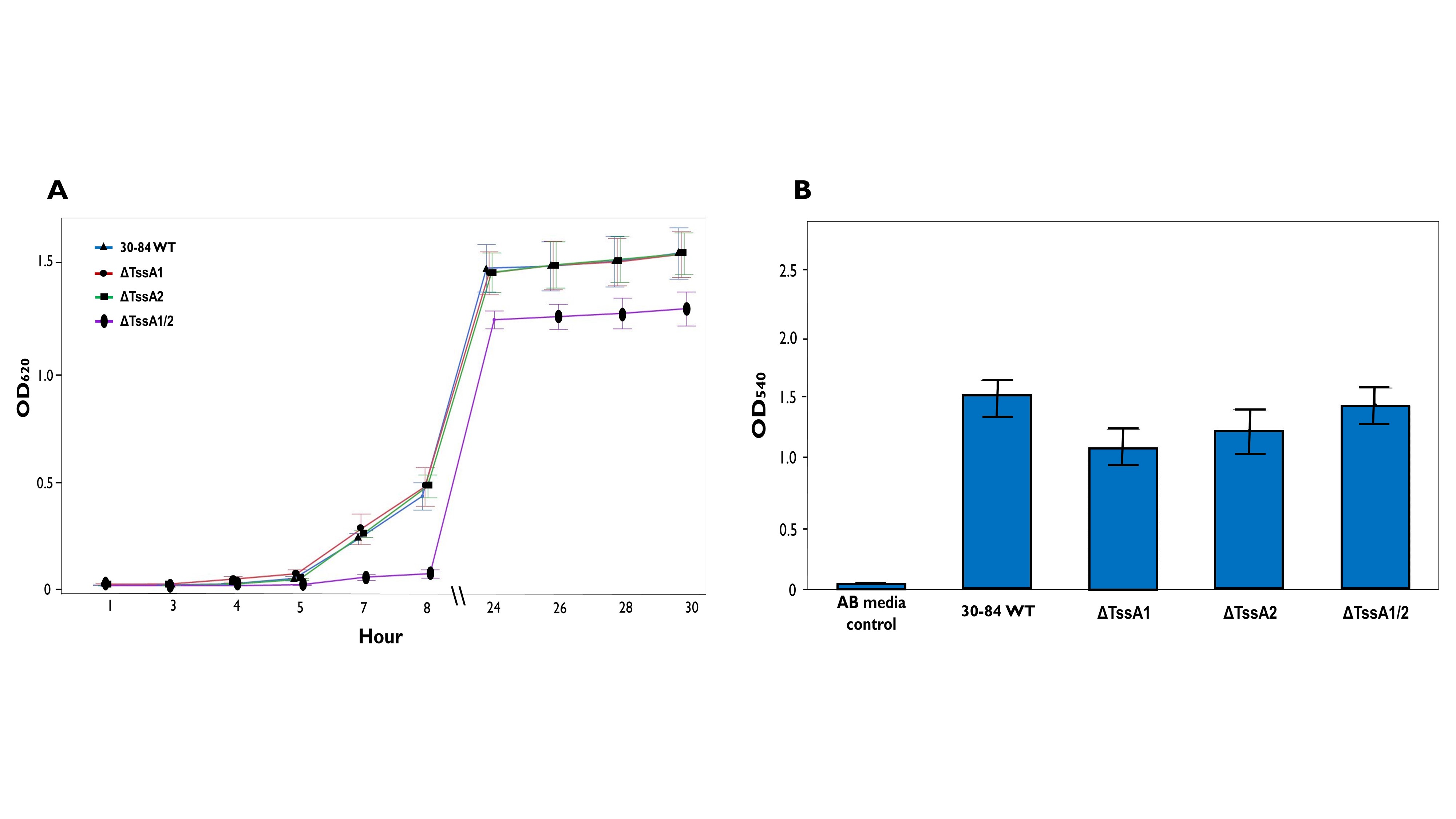

Supplement: Supplementary Figure S2 — Planktonic growth curve and attached biofilm production. (A) Planktonic growth: bacteria were grown in LB medium at 28°C with agitation and populations were measured (OD620) every hour up to 8 h and then every 2 h from 24 h to 30 hours. Data are the mean and standard error of six replicates. (B) Attached biofilms: separate wells of 96-well plates were inoculated with bacteria and grown at 28°C without agitation for 72 h. After removal of non-adhering cells, surface-attached biofilms were stained with crystal violet. The optical density (540 nm) of crystal violet released from the biofilms was used as a relative measure of biofilm population density. Data are the means and standard errors of two biological replicates per strain (started from separate colonies) and five technical replicates performed three times. Strains tested included 30–84 WT, the single T6SS mutants ΔTssA1 and ΔTssA2, and the double mutant ΔTssA1/2. Data were analyzed using a one-way ANOVA and Tukey’s tests, p < 0.05. No significant differences were found. [file Image_2.JPEG]

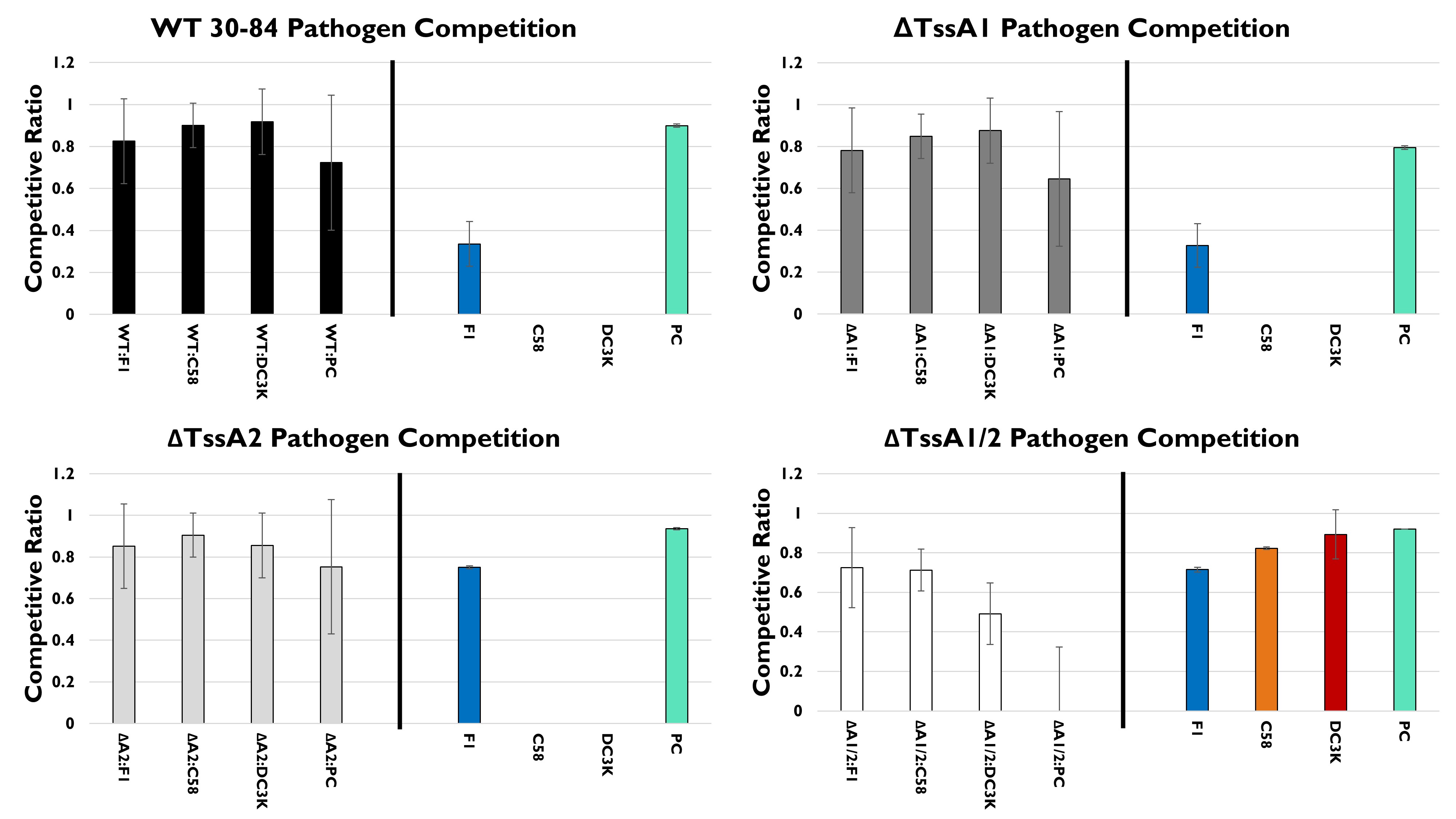

Supplement: Supplementary Figure S3 — In vitro competition assays against environmental isolates and pathogens. The competitive fitness of 30–84 WT and T6SS mutants against environmental isolates and pathogenic strains were evaluated by comparing their populations when grown separately or in 50:50 mixtures in liquid medium. Data are expressed as competitive ratios (population in mixture/population when grown separately). Gray scale bars (left) indicate performance of 30–84WT and derivatives, and colored bars (right) indicate performance of competitors, including P. putida F1 (blue), Agrobacterium tumefaciens C58 (orange), P. syringae DC3000 (red), and Pectobacterium carotovorum (green). Individual bacterial cultures or mixed cultures were spotted onto nitrocellulose filters on LB plates and incubated at 28°C, 5 h. Bacterial cells were washed from filters, collected via centrifugation, and populations were enumerated after 48 h via serial dilution plating. Data are the means (log10 CFU/1 mL) of at least five biological replicates/treatment pooled across at least two experiments and standard errors are indicated. [file Image_3.JPEG]

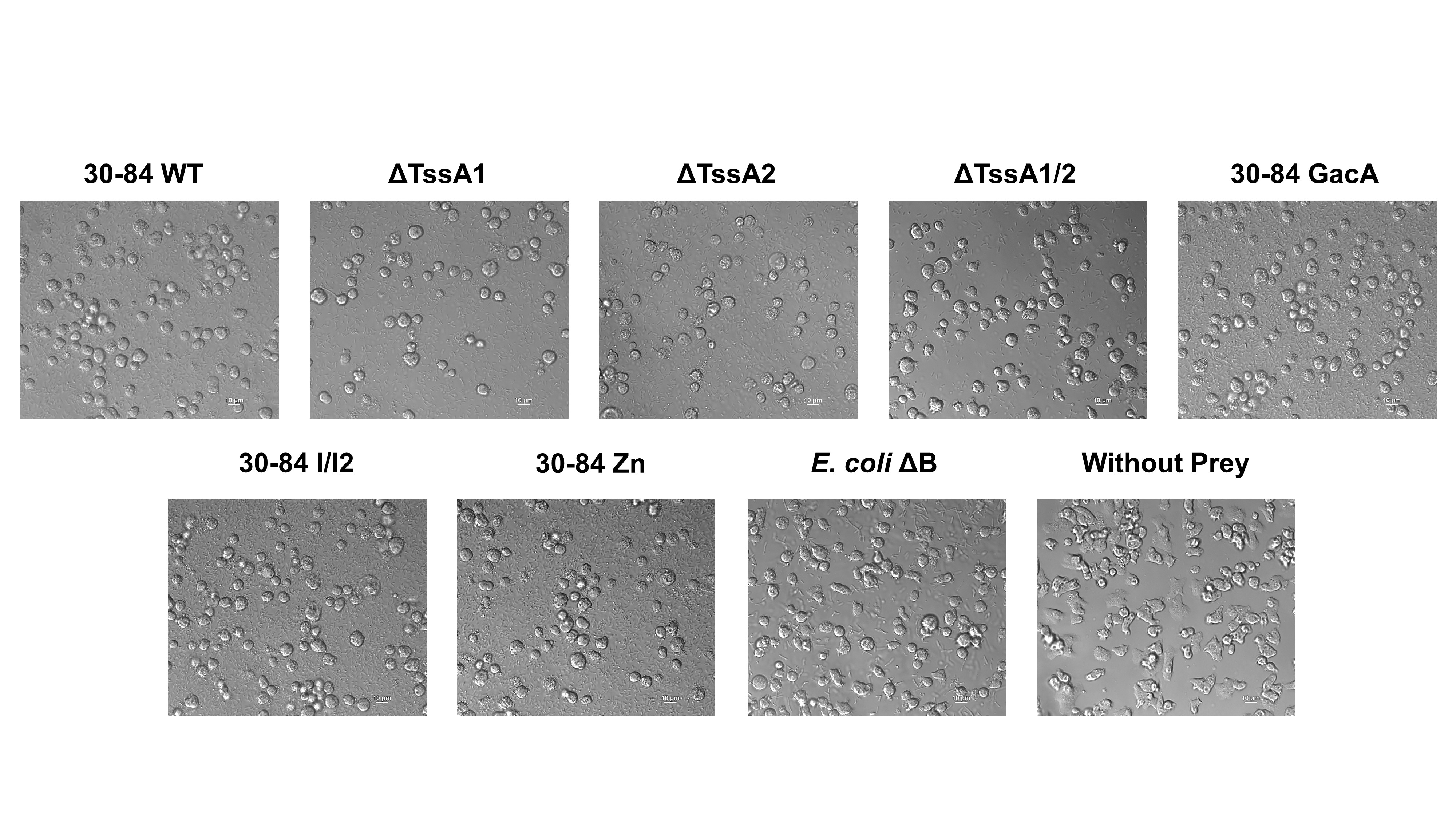

Supplement: Supplementary Figure S4 — Aggregation behavior of Dictyostelium discoideum grown with different bacterial strains in high nutrition HL5 medium. Bacteria used as prey in the feeding assay included 30–84 WT, ΔTssA1, ΔTssA2, ΔTssA1/2, 30–84 GacA, 30–84 I/I2, or 30–84 ZN and E. coliΔB (used as a preferred prey in the lab). D. discoideum without prey bacteria was used as a negative control. D. discoideum cells were grown in 24 well plates in high nutrient HL5 media for 24 h and aggregation behavior was observed using DIC microscopy (100X oil). All treatments showed no aggregation behavior, indicating no stress. Two replicate experiments were performed, and representative images are presented from the same replicate are presented. [file Image_4.JPEG]

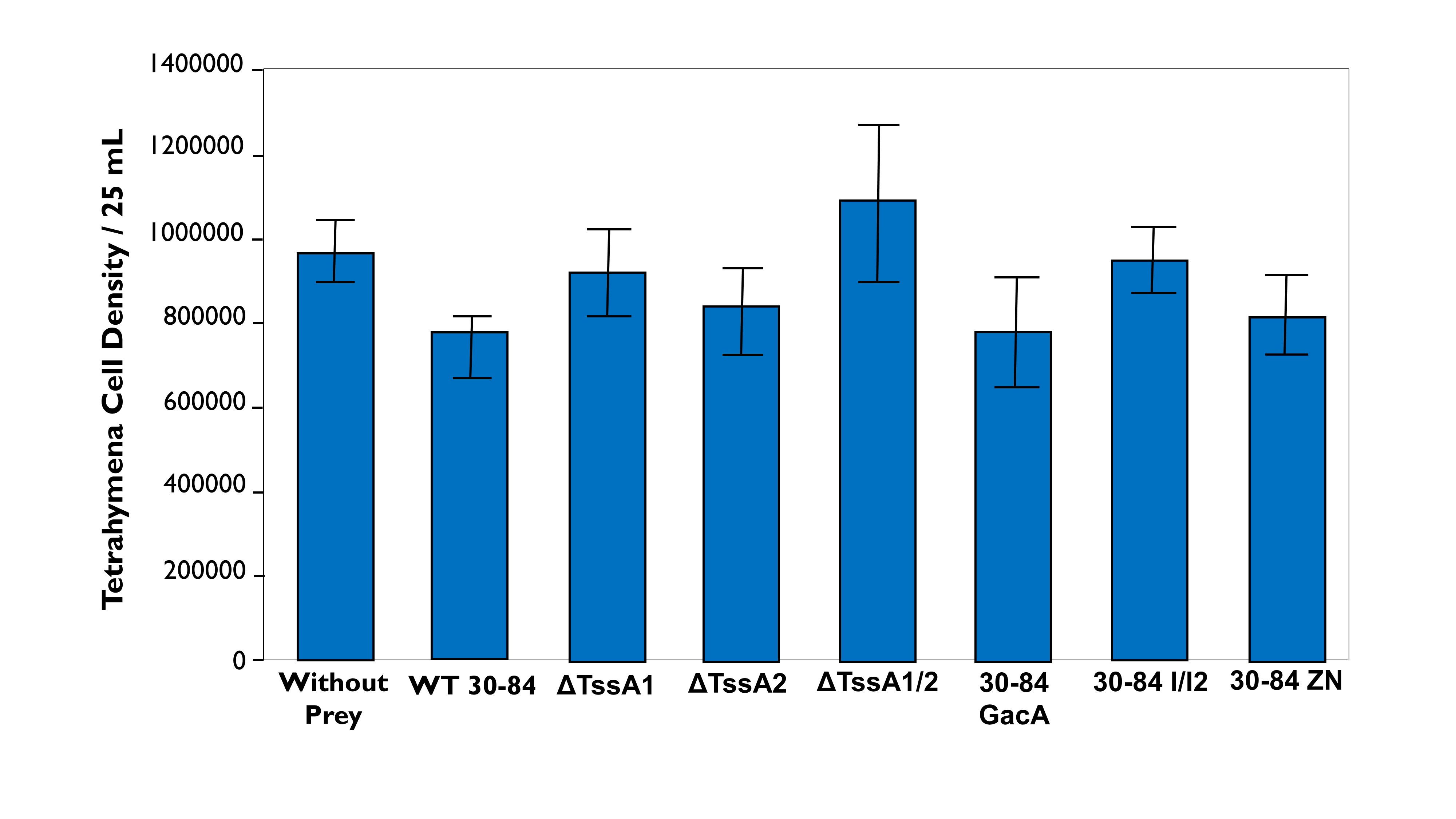

Supplement: Supplementary Figure S5 — Tetrahymena populations after 24 h feeding assay. Bacteria used as prey in the feeding assay included 30–84 WT, ΔTssA1, ΔTssA2, ΔTssA1/2, 30–84 GacA, 30–84 I/I2, or 30–84 ZN. T. thermophila without prey bacteria was used as a negative control. Bacterial and T. thermophila cultures were mixed (5 mL, 20 mL, respectively) and grown in 50 ml tubes with agitation (200 rpm, 27°C). After 24 h, T. thermophila populations in mixed cultures were enumerated via direct counts using a hemocytometer. Data are the means and standard errors of six replicate experiments. Data were analyzed using a one-way ANOVA and Student t-tests (p < 0.05). No significant differences were found. [file Image_5.JPEG]

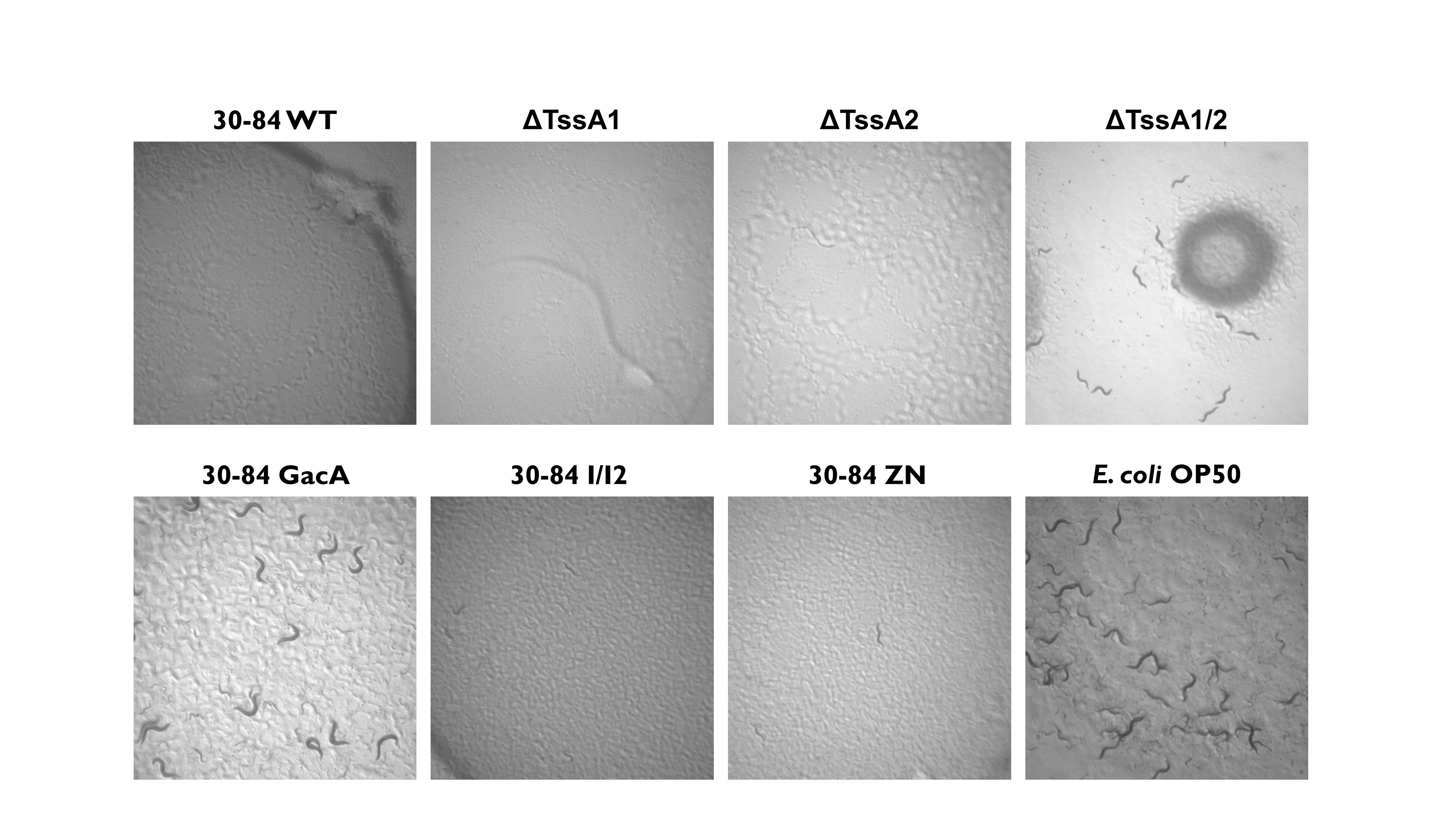

Supplement: Supplementary Figure S6 — Images of C. elegans on plates containing different prey after 72 h. Five adult C. elegans were transferred at 1-hour intervals to new prey-containing plates to facilitate egg laying (a total of four successive transfers), and then images were taken at 72 h. Images are of the center of the plate where bacterial cultures were applied. Plates contained either 30–84 WT, ΔTssA1, ΔTssA2, ΔTssA1/2, 30–84 GacA, 30–84 I/I2, 30–84 ZN, or E. coli OP50 (control) as a food source. Images were obtained using a Zeiss Stemi SV11 scope (26X magnification) and a Hamamatsu ImagEM EM-CCD camera. Adult nematodes can be seen on the plates containing ΔTssA1/2, 30-84 GacA or E. coli OP50 (ΔTssA1/2 image contains air bubble), whereas no or only a few, immature nematodes can be seen on plates with the other prey sources. The experiment and imaging were repeated three times with four plates/replicate (n = 12) and representative images from the same replicate are shown. [file Image_6.JPEG]
