# Supplementary material for: The Type VI Secretion Systems in Plant-Beneficial Bacteria Modulate Prokaryotic and Eukaryotic Interactions in the Rhizosphere
Source: Front Microbiol. 2022 Apr 7;13:843092. doi: 10.3389/fmicb.2022.843092 (PMC9022076; doi:10.3389/fmicb.2022.843092)
Supplement: Supplementary file 7 [file Table_1.DOCX]

**Supplementary Table S1: Primers used in this study**

| **Primers** | |
| --- | --- |
| TssA1KO-repliQa-UP-F | TTTGAGACACAACGTGGCTTTCCCCAGCTGTAGCCCTTGCACCA |
| TssA1KO-repliQa-UP-R | GACCATGATTACGAATTCGAGCTCGCAGGCGGGCGAAAG |
| TssA1KO-repliQa-DWN-F | GCAGGTCGACTCTAGAGGATCCCCGGGTACAGGGTCTTGTGCAGGTC |
| TssA1KO-repliQa-DWN-R | GAGCATTACGCTGACTTGACGGGACACGAGCCCTCAAGCCC |
| TssA1KO-repliQa-KmR-F | ACCGGCACGGGGCTTGAGGGCTCGTGTCCCGTCAAGTCAGCGTAAT |
| TssA1KO-repliQa-KmR-R | CCGGCCTGGTGCAAGGGCTACAGCTGGGGAAAGCCACGTTGTGTC |
| TssA2KO-UP-F-EcoRI | GAATTCCCCGTCTGCCGCAGAGTAGAGCCTG |
| TssA2KO-UP-R-KpnI | CCTATTCGAGGGTACCGTAGGACATCCATGGCCTTTTTTG |
| TssA2KO-DWN-F-KpnI | GATGTCCTACGGTACCCTCGAATAGGCCCCAGGGCCAACA |
| TssA2KO-DWN-R-HindIII | CCCAAGCTTGCGGTGTAGATGTGCTTGTACAGGCC |
| KanR-F-BamHI | GGATCCTAGAAAAACTCATCGAGCATCAAATGAAACTG |
| KanR-R-BamHI | CCTAGGATGAGCCATATTCAACGGGAAACG |
| DoubleKO-GnR+A2UP-R | AAGGCCATGGATGTCCTACCGGTACTCTTACTGTCATGCCATCC |
| DoubleKO-GnR+A2DWN-F | TGGGGCCTATTCGAGGGTACGCAACTCGGTCGCCGCATACA |

*Red coloration indicates restriction enzyme sites.*

**Supplementary Table S2. Predation of Bacteria by *C. elegans* after 72 h.** Estimates of the percentage clearing of the bacterial lawn on each plate are made. This experiment was repeated 3 times with 4 plates/treatment/experiment. Final n = 12 per treatment.

| **Prey** | **>90%** | **90-25%** | **<25%** |
| --- | --- | --- | --- |
| OP50 | 8 | 4 | 0 |
| 30-84 WT | 0 | 0 | 12 |
| ΔTssA1 | 0 | 2 | 10 |
| ΔTssA2 | 0 | 1 | 11 |
| ΔTssA1/2 | 7 | 5 | 0 |
| 30-84 GacA | 10 | 2 | 0 |
| 30-84 I/I2 | 0 | 0 | 12 |
| 30-84 ZN | 0 | 0 | 12 |

**Supplementary Table S3:** **Putative effectors of *Pseudomonas chlororaphis* 30-84.** Bolded words indicate structural proteins of the T6SS needle complex. Red text indicates proteins found within the T6SS-1 cluster. Green text indicates proteins found within the T6SS-2 cluster.

| **Locus Tag** | **Putative Effector** | **Predicted Activity*** | **Putative Localization** |
| --- | --- | --- | --- |
| PCHL3084_RS01885 | Putative RhsP2 | Unknown function; General Antibiotic activity | Cytoplasmic Membrane |
| PCHL3084_RS01890 | Putative Tap (T6SS Adaptor Protein) | Unknown function; DUF4123 domain | Cytoplasmic |
| PCHL3084_RS01895 | **VgrG** | Needle Subunit | Cytoplasmic |
| PCHL3084_RS01900 | **Hcp** | Needle Subunit | Extracellular |
| PCHL3084_RS04425 | **PAAR** protein | Needle Subunit |  |
| PCHL3084_RS04675 | **PAAR** protein | Needle Subunit |  |
| PCHL3084_RS11710 | **PAAR** protein | Needle Subunit |  |
| PCHL3084_RS14860 | Hypothetical protein | peptidoglycan-binding - LysM binding motif |  |
| PCHL3084_RS14865 | Hypothetical protein | DUF4123 domain |  |
| PCHL3084_RS14870 | **VgrG** | Needle Subunit | Cytoplasmic |
| PCHL3084_RS15625 | Hypothetical protein | Putative lipase | Cytoplasmic |
| PCHL3084_RS15635 | **VgrG** | Needle Subunit | Cytoplasmic |
| **PCHL3084_RS17720** | **Hcp** | Needle Subunit | Extracellular |
| **PCHL3084_RS17725** | Putative Tae4 | Amidase activity |  |
| **PCHL3084_RS17730** | Hypothetical protein | Unknown function |  |
| **PCHL3084_RS17755** | **VgrG** | Needle Subunit | Cytoplasmic |
| **PCHL3084_RS17760** | Hypothetical protein | DUF6484 domain | Cytoplasmic |
| **PCHL3084_RS17765** | Hypothetical protein | DUF2169 domain |  |
| PCHL3084_RS17830 | Hypothetical protein | Putative lipase |  |
| PCHL3084_RS17835 | Hypothetical protein | Putative lipoprotein | Cytoplasmic |
| PCHL3084_RS17840 | Hypothetical protein | DUF4123 domain |  |
| PCHL3084_RS17845 | **VgrG** | Needle Subunit | Cytoplasmic |
| PCHL3084_RS18065 | **PAAR** protein | Needle Subunit |  |
| PCHL3084_RS18305 | **PAAR** protein | Needle Subunit |  |
| PCHL3084_RS20050 | **PAAR** protein | Needle Subunit |  |
| PCHL3084_RS20055 | Putative TplEi | TplEi Immunity protein, RS20060 paralog |  |
| PCHL3084_RS20060 | Putative TplEi | TplEi Immunity protein, RS20055 paralog |  |
| PCHL3084_RS20065 | Putative TplE | Phospholipase |  |
| PCHL3084_RS20070 | **VgrG** | Needle Subunit | Cytoplasmic |
| PCHL3084_RS20265 | **VgrG** | Needle Subunit | Cytoplasmic |
| PCHL3084_RS20270 | Hypothetical protein | DUF4123 domain | Cytoplasmic |
| PCHL3084_RS20275 | Hypothetical protein | Putative lipase | Cytoplasmic |
| PCHL3084_RS20575 | Hypothetical protein | (3) Sel1-like repeats |  |
| PCHL3084_RS20580 | Putative Phospholipase D family protein | Phospholipase; Homology to T6SS effector Tle5b |  |
| PCHL3084_RS20585 | **VgrG** | Needle Subunit | Cytoplasmic |
| PCHL3084_RS21180 | **PAAR** protein | Needle Subunit |  |
| PCHL3084_RS24445 | **PAAR** protein | Needle Subunit |  |
| PCHL3084_RS27685 | **PAAR** protein | Needle Subunit |  |
| **PCHL3084_RS29450** | Putative Tle1 | Phospholipase, DUF2235 domain |  |
| **PCHL3084_RS29455** | Putative Tli1 | Tle1 Immunity protein |  |
| **PCHL3084_RS29460** | Hypothetical protein | DUF4123  domain | Cytoplasmic |
| **PCHL3084_RS29465** | **VgrG** | Needle Subunit | Cytoplasmic |
| **PCHL3084_RS29475** | Protein with lysozyme-like domain similar to pesticin | Hydrolysis of peptidoglycan |  |
| **PCHL3084_RS29480** | **VgrG** | Needle Subunit | Cytoplasmic |
| PCHL3084_RS00060 | **PAAR** protein | Needle Subunit |  |

***Predicted Activity**

**RS01885, RS01890, RS01895, and RS01900**: The four gene cluster contains genes encoding a RhsA-like protein, a TAP (T6 adaptor protein) with unknown function, VgrG and Hcp, respectively.

**RS14860, RS14865, and RS14870**: The three gene cluster encodes a hypothetical protein with a peptidoglycan-binding LysM binding motif, a hypothetical protein with a DUF4123 binding domain, and a VgrG-encoding gene, respectively.

**RS15625, RS15635**: Genes encoding a hypothetical protein predicted to have lipase (class 3) activity and VgrG, respectively.

**RS17720, RS17725, and RS17730**: The three gene cluster located within the T6SS-1 cluster encodes Hcp, a Tae4-like protein, and a hypothetical protein (with no homology to an immunity protein), respectively.

**RS17755, RS17760, RS17765**: Also located within the T6SS-1 cluster are genes encoding VgrG and two hypothetical proteins with DUF6484 (unknown function, associated with T6SS) and DUF2169 domains, respectively.

**RS17830, RS17835, RS17840, and RS17845**: Located nearby the T6SS-1 gene cluster are four genes encoding a hypothetical protein predicted to have lipase (class 3) activity, a hypothetical cytoplasm-localized lipoprotein, a hypothetical protein with a DUF4123 domain, and VgrG, respectively.

**RS20050, RS20055, RS20060, RS20065, RS20070**: The five gene cluster encodes a PAAR protein, a pair of putative immunity protein (paralogs), a TplE-like protein and VgrG, respectively.

**RS20265, RS20270, RS20275**: Genes encoding VgrG, a hypothetical protein predicted to have a DUF4123 domain, and a hypothetical protein predicted to have lipase (class 3) activity, respectively.

**RS20575, RS20580 and RS20585**: Genes encoding VgrG, a putative type VI secretion phospholipase D effector (Pld) in the same ortholog group as *tle5B* in *P. aeruginosa* PAO1, and a hypothetical that contains three Sel1-like repeats (SLRs).

**RS29450, RS29455, RS29460, RS29465, RS29475 and RS29480**: Located within the T6SS-2 gene cluster are genes encoding a Tle1-like phospholipase protein with a DUF2235 domain, a Tli1-like immunity protein with a DUF3304 domain, a hypothetical protein with a DUF4123 domain, and VgrG, respectively. Located between the two VgrG-encoding genes (RS29465 and RS29480) is a hypothetical protein having a lysozyme-like domain similar to the C-terminal domain of pesticin (RS29475).
